# Study on the pressure relief energy dissipation law of variable-diameter boreholes in roadway surrounding rock under dynamic and static loads

**DOI:** 10.1371/journal.pone.0306449

**Published:** 2024-09-06

**Authors:** Jinguo Lyu, Linfan Qi, Yishan Pan, Lianpeng Dai, Zhi Tang, Xuebin Wang

**Affiliations:** 1 School of Mechanics and Engineering, Liaoning Technical University, Fuxin, Liaoning, China; 2 School of Physics, Liaoning University, Shenyang, Liaoning, China; 3 Institute of Disaster Rock Mechanics, Liaoning University, Shenyang, Liaoning, China; University of Vigo, SPAIN

## Abstract

To address the conflict between pressure relief and support effectiveness caused by large-diameter boreholes in roadway surrounding rock, this paper proposes a method involving variable-diameter boreholes for pressure relief and energy dissipation. With a typical rock burst coal mine as the engineering context, the study establishes a mechanical model for variable-diameter boreholes through theoretical analysis to examine the elastic stress distribution around boreholes within the coal body. Physical similarity simulation tests are conducted to investigate the influence of conventional borehole and variable diameter borehole on the transmission pattern of dynamic load stress waves. Furthermore, numerical simulations are employed to explore the effects of reaming diameter, depth, and spacing on pressure relief, energy dissipation, and attenuation of dynamic stress wave transmission in roadway surrounding rock. The results demonstrate that stress within the coal surrounding the variable-diameter borehole correlates with the borehole radius, lateral pressure coefficient, and distance from the point to the borehole center, the extent of the plastic zone is influenced by borehole diameter, spacing, and depth. Increased diameter, reduced spacing, and greater depth of deep reaming holes exacerbate the transfer of stress concentration from the surrounding rock of the roadway to the deeper regions, facilitating the formation of stress double peak areas. Moreover, the variable diameter position should be within the original stress peak position of the surrounding rock in the roadway, with deep reaming passing through the stress concentration area for optimal results. This study offers guidance on the prevention and control technology for rock bursts in deep coal mining operations.

## 1. Introduction

Pressure bumping represents one of the most severe dynamic hazards in deep coal mining operations in China [1–5]. As coal mining in the country deepens and intensifies, the frequency and severity of rock bursts are also on the rise. Pressure bumping events result in the rapid release of internally stored strain energy, causing significant dislodgement of surrounding coal rock from the parent rock body. This phenomenon often leads to bracket failure and major casualty accidents. Currently, methods for preventing and controlling rock bursts in coal mines include coal seam water injection, coal seam blasting, roof and bottom breaking blasting, and borehole for pressure relief [6–9]. Among these methods, borehole for pressure relief stands out due to its simple construction technology, low cost, minimal impact on production efficiency, and ease of operation. Consequently, it has become widely adopted for rock burst prevention and control.

The design of parameters is the most crucial aspect of borehole pressure relief [7, 10–15]. Excessive pressure relief can result from overly dense borehole arrangements, adversely affecting support and escalating production costs. Conversely, overly sparse borehole layouts can lead to inadequate pressure relief, resulting in new stress concentrations in the surrounding rock of the roadway and heightening the risk of coal body impacts. Yao, JP et al. [13] integrated the mechanism of borehole pressure relief with roadway support, investigating the energy characteristics of the anchorage strengthening zone and stress reduction zone. They suggested positioning the borehole between the anchorage end and the peak stress. Li et al. [14] analyzed elastic strain. The elastic-plastic state of circular boreholes in elastic strain-softening coal seams was analyzed. P. N. Tambovtsev [15] established a similar analytical mechanical model to analyze the energy input required to generate macroscopic cracks under different borehole diameters. Zhang et al. [7] studied the changes of uniaxial compressive strength and energy dissipation index of coal after drilling, and analyzed the characteristics of crack propagation in coal. Lan Yongwei [16] concluded through numerical simulation that, under constant conditions, the decrease in coal seam strength or increase in coal seam pressure leads to an expansion in the failure radius of coal around the borehole. He attributed this phenomenon to coal softening around the borehole, which is the root cause of pressure relief. Wang Aiwen et al. [17, 18] investigated the influence of borehole number and arrangement on the mechanical and impact performance parameters of samples. They observed that an increase in borehole number led to gradual decreases in elastic modulus, stress peak, and impact energy index, resulting in improved pressure relief effect. Li Yuewen et al. [19] simulated the impact of borehole diameter and spacing on borehole pressure relief under varying burial depths using FLAC3D software. The results revealed concentrated stress on the sides and formation of stress reduction zones above and below the borehole. Larger borehole diameters result in improved pressure relief effects, while smaller borehole spacings also enhance pressure relief effects. Combined with theoretical analysis, FLAC3D numerical simulation and field monitoring, Gao Yongge [20] deeply explored the evolution law of stress distribution around boreholes with different apertures under the same geological conditions. It is found that under the same geological conditions, a moderate increase in the diameter of the borehole can significantly increase the radius of the plastic zone around the borehole and effectively improve the pressure relief effect of the borehole.

Domestic and international scholars have conducted extensive research on the stress and energy release of roadway surrounding rock using conventional borehole parameters, yielding significant insights for underground rock burst prevention and control. However, conventional boreholes have limited pressure relief effectiveness and stress transfer capability on roadway surrounding rock. Moreover, excessive borehole diameter can jeopardize support structures, creating a conflict with surrounding rock control. Effectively conducting borehole pressure relief in high-risk areas without compromising roadway support strength is pivotal for coordinated rock burst disaster management and roadway control. To address this challenge, this study proposes variable diameter borehole based on conventional methods. Small diameter borehole is employed alongside the roadway, while large diameter reaming is conducted within the deeper layers of surrounding rock. Subsequently, boreholes in the roadway anchorage zone are sealed post-borehole to mitigate their impact on support structures. This approach offers insights for optimizing pressure relief borehole parameters and guiding engineering practices.

## 2. Stress distribution characteristics of coal surrounding variable-diameter boreholes

### 2.1 Variable diameter boreholes mechanical model

**Fig 1:**
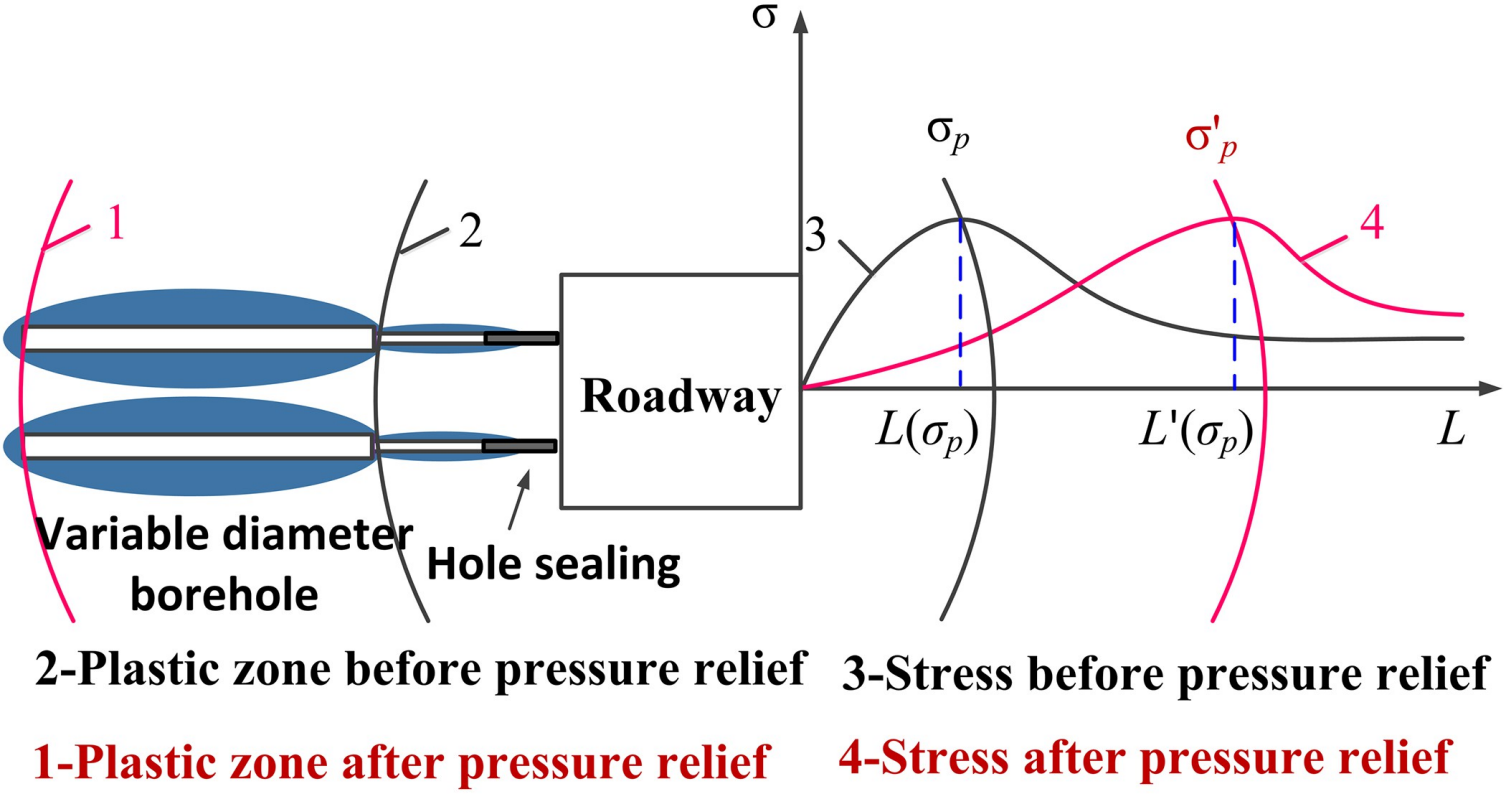
Pressure relief principle diagram of variable aperture borehole.

**Fig 2:**
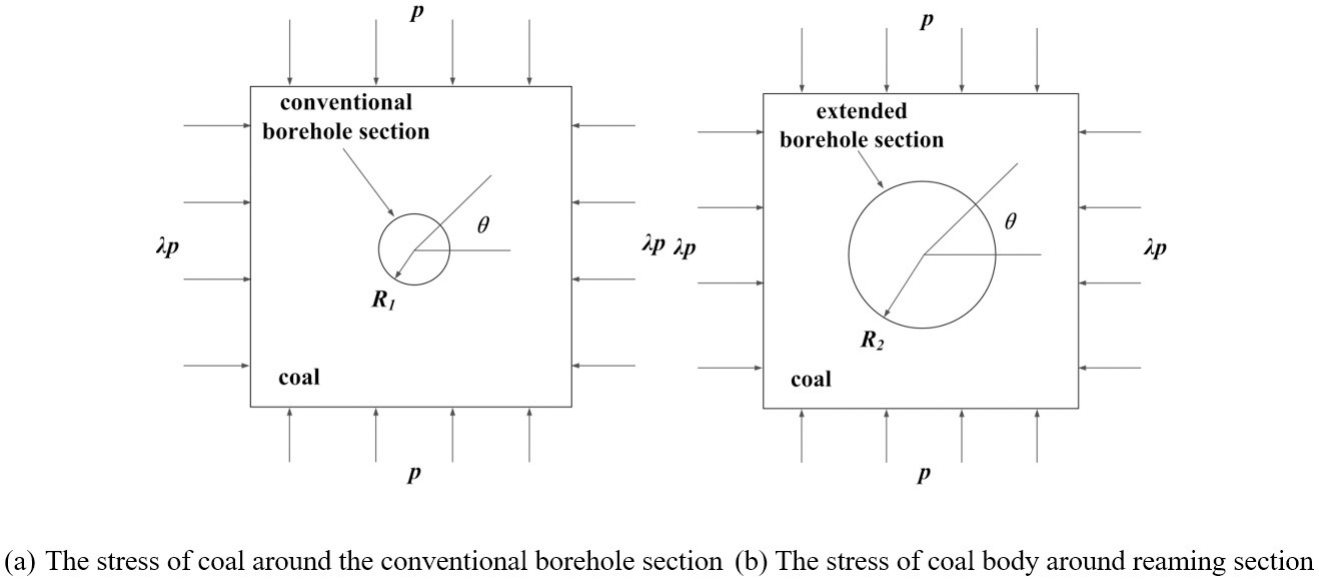
Principle diagram of coal body stress model around borehole.

Scholars, both domestically and internationally, have extensively explored the theoretical derivations regarding the stress distribution and plastic zone in the surrounding rock of circular holes. Building upon prior studies, this research employs elastic-plastic mechanics theory to analyze the stress distribution characteristics of the surrounding rock around segmented reaming. Fig 1 illustrates the schematic diagram of variable diameter borehole. Utilizing elastic mechanics theory, the stress problem concerning borehole surrounding rock is simplified as a plane strain problem for analysis, with uniform stress assumed along both vertical and horizontal directions. The stress state of borehole surrounding rock with variable diameter is depicted in Fig 2.

### 2.2 Elastic solution for stress distribution around boreholes in coal

**Fig 3:**
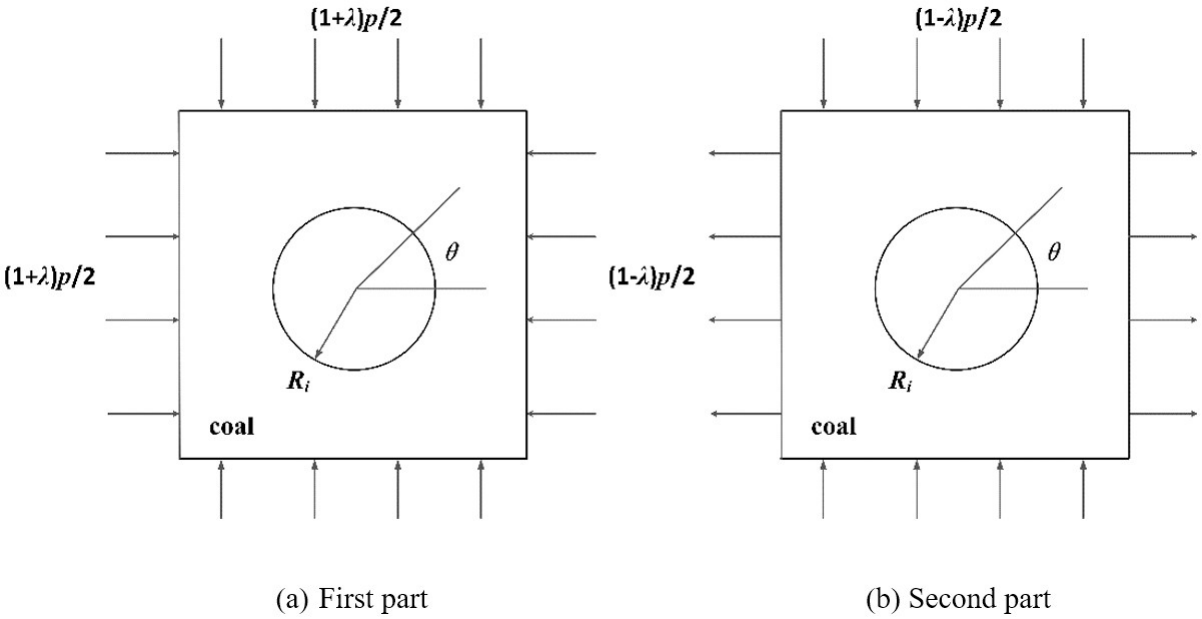
Principle diagram of coal body stress model around borehole.

According to the superposition principle of elastic mechanics, the vertical stress and horizontal stress of the conventional borehole section and the deep reaming section can be decomposed into two parts: the first part is affected by the uniform compressive stress (1 + *λ*)*p*/2 in the vertical direction and the uniform compressive stress (1 + *λ*)*p*/2 in the horizontal direction, as shown in Fig 3(a). The second part is affected by the uniform compressive stress (1 − *λ*)*p*/2 in the vertical direction and the uniform tensile stress (1 − *λ*)*p*/2 in the horizontal direction, as shown in Fig 3(b). By superimposing the two stress conditions, the stress component under the stress condition shown in Fig 2 can be obtained.

First of all, for the stress situation of the first part of Fig 3, according to the theory of elastic mechanics, the equivalent ring can be subjected to uniform pressure (1 + *λ*)*p*/2, and the stress component of the rock mass around the borehole in polar coordinates is deduced as follows:

$$\left\{\begin{array}{l} \sigma_{r}=-\frac{\left(1+\lambda \right)p}{2}\left(1-\frac{a^{2}}{r^{2}}\right)\\ \sigma_{\theta }=-\frac{(1+\lambda )p}{2}\left(1+\frac{a^{2}}{r^{2}}\right)\\ \tau_{r\theta }=0 \end{array}\right.$$ (1)

In the formula: *a* is the radius of the borehole; *r* is the distance from any point in the surrounding rock mass to the center of the borehole.

Secondly, for the stress situation in the second part of Fig 3, according to the classical solution of elastic mechanics, the stress components of the coal body around the conventional borehole section and the deep reaming section can be obtained as follows:

$$\left\{\begin{array}{l} \sigma_{r}=\frac{\left(1-\lambda \right)p}{2}\left(1-\frac{a^{2}}{r^{2}}\right)\left(1-3\frac{a^{2}}{r^{2}}\right)\text{cos}\;2\theta \\ \sigma_{\theta }=-\frac{\left(1-\lambda \right)p}{2}\left(1+3\frac{a^{2}}{r^{2}}\right)\text{cos}\;2\theta \\ \tau_{r\theta }=-\frac{\left(1-\lambda \right)p}{2}\left(1-\frac{a^{2}}{r^{2}}\right)\left(1+3\frac{a^{2}}{r^{2}}\right)\text{sin}\;2\theta \end{array}\right.$$ (2)

By adding the stress components of the first part and the second part, the polar coordinate stress component expression under the action of stress in Fig 2 is obtained:

$$\left\{\begin{array}{l} \sigma_{r}=-\frac{\left(1+\lambda \right)p}{2}\left(1-\frac{a^{2}}{r^{2}}\right)+\frac{\left(1-\lambda \right)p}{2}\left(1-\frac{a^{2}}{r^{2}}\right)\left(1-3\frac{a^{2}}{r^{2}}\right)\text{cos}\;2\theta \\ \sigma_{\theta }=-\frac{\left(1+\lambda \right)p}{2}\left(1+\frac{a^{2}}{r^{2}}\right)-\frac{\left(1-\lambda \right)p}{2}\left(1+3\frac{a^{2}}{r^{2}}\right)\text{cos}\;2\theta \\ \tau_{r\theta }=-\frac{\left(1-\lambda \right)p}{2}\left(1-\frac{a^{2}}{r^{2}}\right)\left(1+3\frac{a^{2}}{r^{2}}\right)\text{sin}\;2\theta \end{array}\right.$$ (3)

In the formula: *r*, *θ* are the polar coordinates of any point around the borehole, *σ*_*r*_ is the radial stress at any point around the borehole, *σ*_*θ*_ is the tangential stress at any point around the borehole, *τ*_*rθ*_ is the shear stress at any point around the borehole, *λ* is the lateral pressure coefficient, *a* is the borehole radius.

### 2.3 Calculation of distribution radius of coal pressure relief zone around boreholes

According to the stress component Eq (3) in polar coordinates, the expression of the principal stress at a point of coal around the borehole is obtained:

$$\left\{\begin{array}{l} \sigma_{1}=\frac{\sigma_{p}+\sigma_{\varphi }}{2}+\frac{1}{2}\sqrt{{\left(\sigma_{p}-\sigma_{\varphi }\right)}^{2}+4\tau_{\rho \varphi }^{2}}\\ \sigma_{3}=\frac{\sigma_{p}+\sigma_{\varphi }}{2}-\frac{1}{2}\sqrt{{\left(\sigma_{p}-\sigma_{\varphi }\right)}^{2}+4\tau_{\rho \varphi }^{2}} \end{array}\right.$$ (4)

In the formula:, are the maximum and minimum principal stress at a point.

According to the principal stress at a point of coal around the borehole, the Mohr-Coulomb yield condition is substituted into Eq (4) to obtain the yield condition at a point of coal around the borehole:

$$\frac{1}{2}\sqrt{{\left(\sigma_{p}-\sigma_{\varphi }\right)}^{2}+4\tau_{\rho \varphi }^{2}}=C\;\text{cos}\;\phi -\frac{1}{2}\left(\sigma_{\rho }+\sigma_{\varphi }\right)\text{sin}\;\phi$$ (5)

In the formula: *C* is the cohesion of coal body, *ϕ* is the internal friction angle of coal.

The boundary equation of the plastic zone of the coal body around the borehole, that is, the pressure relief zone, is derived by substituting the formula (3) into the formula (5):

$$b+h\frac{\rho^{2}}{a^{2}}+d\frac{\rho^{4}}{a^{4}}+e\frac{\rho^{6}}{a^{6}}+f\frac{\rho^{8}}{a^{8}}=0$$ (6)

In the formula: *b* = 9(1−*γ*)^2^

$$h=6\left(1-\gamma^{2}\right)\text{cos}\;2\varphi -12{\left(1-\gamma \right)}^{2}$$

$$\begin{array}{c} d={\left(1+\gamma \right)}^{2}+10{\left(1-\gamma \right)}^{2}{\text{cos}}^{2}\;2\varphi -2{\left(1-\gamma \right)}^{2}{\text{sin}}^{2}\;2\varphi -4\left(1-\gamma^{2}\right)\text{cos}\;2\varphi \\ -4{\left(1-\gamma \right)}^{2}{\text{cos}}^{2}\;2\varphi \cdot {\text{sin}}^{2}\;\phi \end{array}$$

$$\begin{array}{c} e=4{\left(1-\gamma \right)}^{2}\left({\text{sin}}^{2}\;2\varphi -{\text{cos}}^{2}\;2\varphi \right)+2\left(1-\gamma^{2}\right)\text{cos}\;2\varphi \\ -4\frac{C}{p}\left(1-\gamma \right)\text{sin}\;2\phi \;\text{cos}\;2\varphi -4\left(1-\gamma^{2}\right)\text{cos}\;2\varphi \cdot {\text{sin}}^{2}\;\phi \end{array}$$

$$f={\left(1-\gamma \right)}^{2}-4\frac{C^{2}}{p^{2}}{\text{cos}}^{2}\;\phi -{\left(1+\gamma \right)}^{2}{\text{sin}}^{2}\;\phi -2\frac{C}{p}\left(1+\gamma \right)\text{sin}\;2\phi$$

*ρ* is the radius of the pressure relief zone at the corresponding corner *φ*.

Eq (6) reveals that the extent of the pressure relief zone surrounding the borehole depends on the vertical stress *p*, lateral pressure coefficient *γ*, borehole radius *a*, coal cohesion *C*, and internal friction angle *ϕ*.

The analysis indicates that several factors influence the distribution of the coal pressure relief zone around the borehole, including vertical stress *p*, lateral pressure coefficient *γ*, borehole radius *a*, coal cohesion *C*, and internal friction angle *ϕ*. Among them, the cohesion *C*, internal friction angle *ϕ*, and lateral pressure coefficient *γ* of the coal body are determined by the working face environment, while the diameter of conventional drilling and deep reaming, as well as the spacing of drilling, determine their size *a*. Additionally, excavation affects the vertical stress of coal and surrounding rock near the borehole, resulting in varying stress *p* states of the coal surrounding the borehole due to differences in borehole depth. Consequently, the borehole depth determines the borehole stress, consequently influencing the distribution of the coal’s pressure relief area around the borehole. It follows that the pressure relief effectiveness of variable diameter boreholes and the radius of the surrounding coal body’s plastic zone are influenced by the diameters of conventional borehole sections, deep reaming sections, borehole spacing, and borehole depth.

## 3. Numerical simulation

### 3.1 Scheme of numerical simulation

In contrast to conventional borehole pressure relief, variable-diameter borehole pressure relief involves additional influencing factors, primarily deep borehole diameter, variable aperture position, and borehole spacing. To examine the impact of these three key parameters on the stability of roadway surrounding rock, FLAC3D numerical simulation software was utilized to analyze the vertical stress distribution and energy dissipation characteristics of various variable aperture borehole parameters based on engineering context.

**Fig 4:**
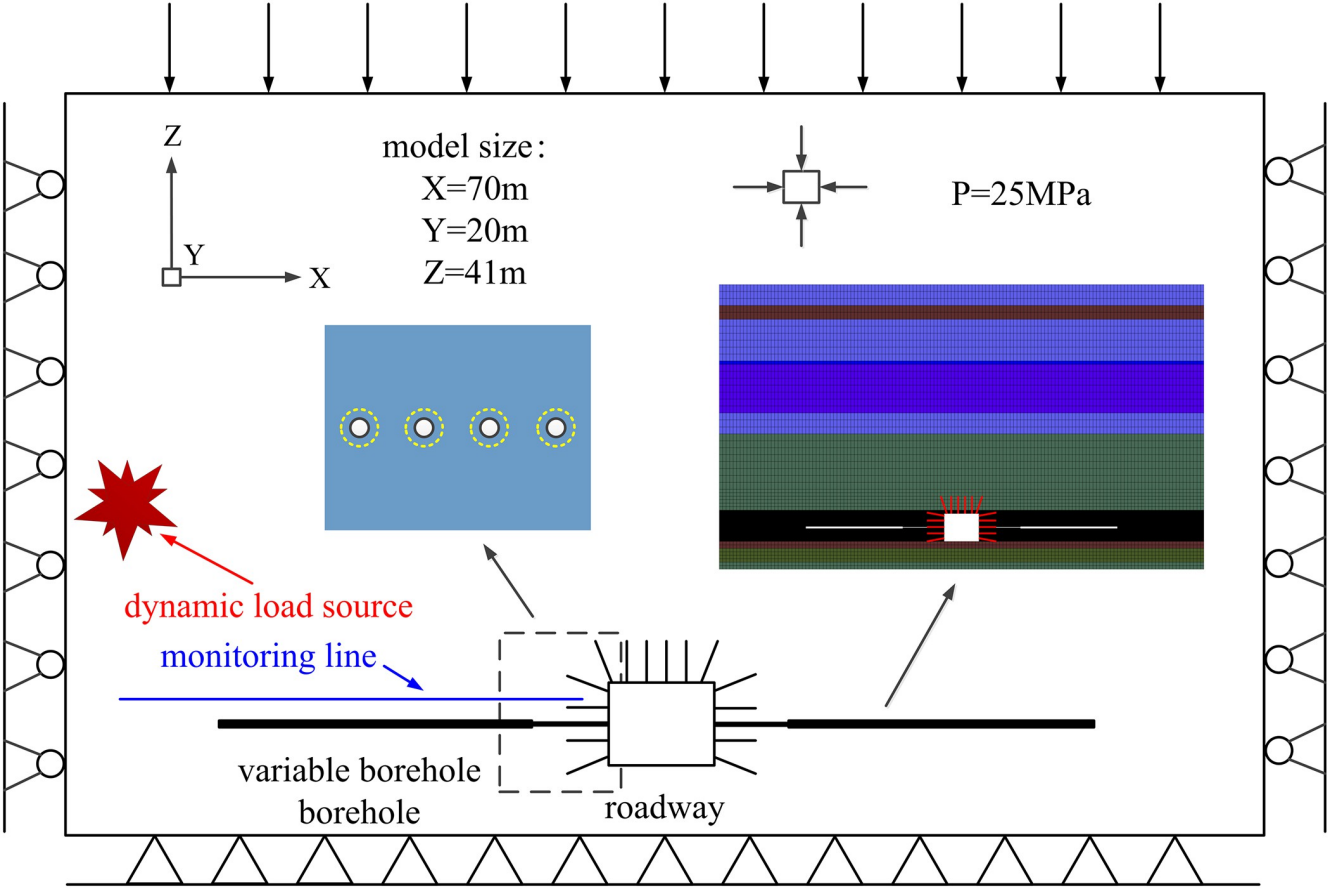
Variable diameter borehole numerical simulation model diagram.

A three-dimensional numerical model is constructed for representative rock burst coal mines, with dimensions of 70 m × 20 m × 41 m (length, width, and height). Horizontal displacement constraints are enforced on the left, right, front, and rear boundaries, while the bottom boundary remains fixed. Although the model’s buried depth does not reflect the actual depth, the remaining depth is equivalently simulated by applying stress from above. The true buried depth is approximately 1000 m, with self-weight stress being the primary stress due to the simple geological structure. A uniform load of 25.0 MPa is applied to the model’s top, based on practical experience and mining theory. During dynamic calculations, static boundaries are used for the top and bottom of the model, while free field boundary conditions are applied to the front, back, left, and right boundaries. This approach minimizes wave reflection on the coal-rock boundary and simulates an infinite site effect. The distribution of mine earthquakes on the impact day and the spatial coordinates and energy of monitored significant mine earthquake events guide the model setup. The Dynamic module of FLAC3D software is utilized to apply a simple harmonic stress wave, simulating a 10^5^ J mine earthquake occurring 20 m above the roadway. The source strength is set to 40 MPa with a frequency of 100 Hz. The simulated dynamic load time spans one period (0.02 s), while the dynamic load action time is 0.1 s. A monitoring line, comprising 26 stress measuring points spaced 1.0 m apart and 33 acceleration measuring points spaced 1.0 m apart, is arranged around the roadway to track stress and acceleration changes in the surrounding rock. To better align the simulation environment with the actual site conditions, Rayleigh damping parameters are introduced to allow gradual energy dissipation with the expansion of the stress-strain hysteresis loop. Mesh units in the roadway excavation support area are densely divided, while those in other areas are relatively sparse. Hexahedral elements are used for the grid, and a representative three-dimensional numerical model is selected, as depicted in Fig 4.

**Scheme 1.** The conventional shallow borehole has a fixed diameter of 100 mm and a depth of 4 m. The deep reaming extends to a depth of 12 m, with a borehole spacing of 1.0 m. The total length of the borehole is 20 m. The scheme is shown in Table 1.

**Table 1:** Numerical simulation schemes of different deep reaming diameters.

Scheme	1	2	3	4	5
Deep reaming diameter (mm)	100	150	200	250	300
Shallow borehole diameter (mm)	100	100	100	100	100
Shallow borehole depth (m)	4	4	4	4	4
Deep reaming depth (m)	16	16	16	16	16
Borehole spacing (m)	1.0	1.0	1.0	1.0	1.0

**Scheme 2.** The conventional shallow borehole has a fixed diameter of 100 mm, while the deep reaming borehole measures 250 mm in diameter. Both boreholes are spaced at intervals of 1.0 m along a total length of 20 m. The scheme is shown in Table 2.

**Table 2:** Numerical simulation schemes of different deep reaming depth.

Scheme	1	2	3	4	5	6
Shallow borehole depth (m)	0	4	8	12	16	20
Deep reaming depth (m)	20	16	12	8	4	0
Shallow borehole diameter (mm)	100	100	100	100	100	100
Deep reaming diameter (mm)	250	250	250	250	250	250
Borehole spacing (m)	1.0	1.0	1.0	1.0	1.0	1.0

**Scheme 3.** The conventional shallow borehole has a diameter of 100 mm, while the deep reaming borehole has a diameter of 250 mm. The shallow borehole extends to a depth of 4 m, whereas the deep reaming reaches a depth of 16 m. Both boreholes span a total length of 20 m. The scheme is shown in Table 3.

**Table 3:** Numerical simulation scheme of different deep reaming spacing.

Scheme	1	2	3	4	5
Borehole spacing (m)	0.5	1.0	1.5	2.0	2.5
Shallow borehole diameter (mm)	100	100	100	100	100
Deep reaming diameter (mm)	250	250	250	250	250
Shallow borehole depth (m)	4	4	4	4	4
Deep reaming depth (m)	16	16	16	16	16

### 3.2 Numerical simulation results analysis

#### 3.2.1 Numerical simulation schemes of different deep reaming diameters

**Fig 5:**
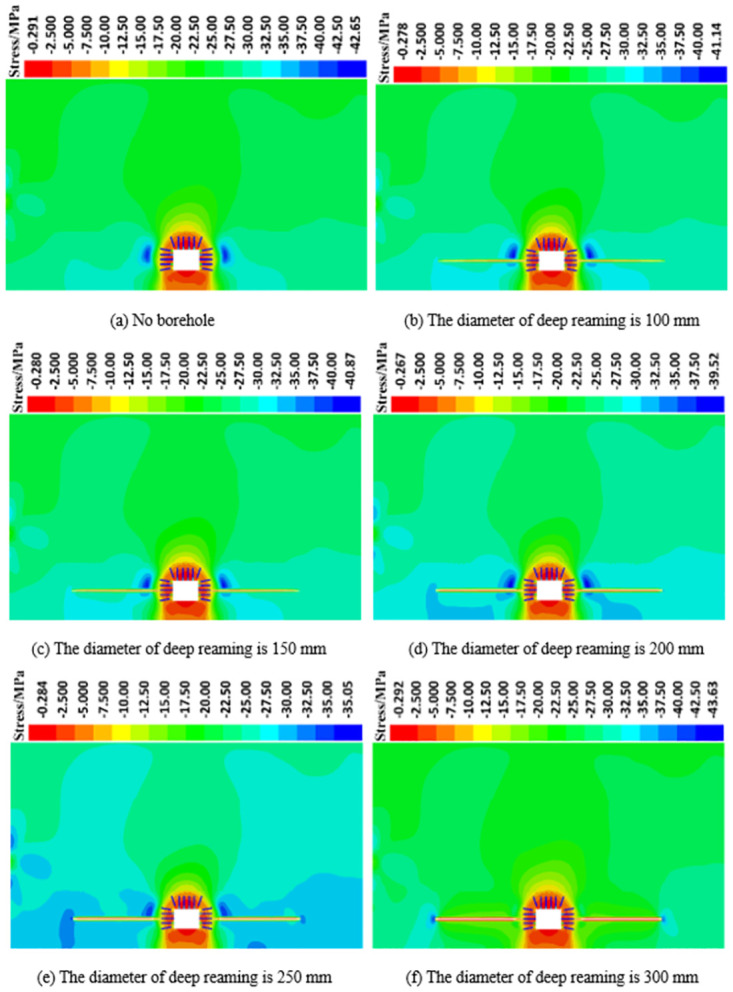
Vertical stress distribution of roadway surrounding rock under different deep reaming diameters.

**Fig 6:**
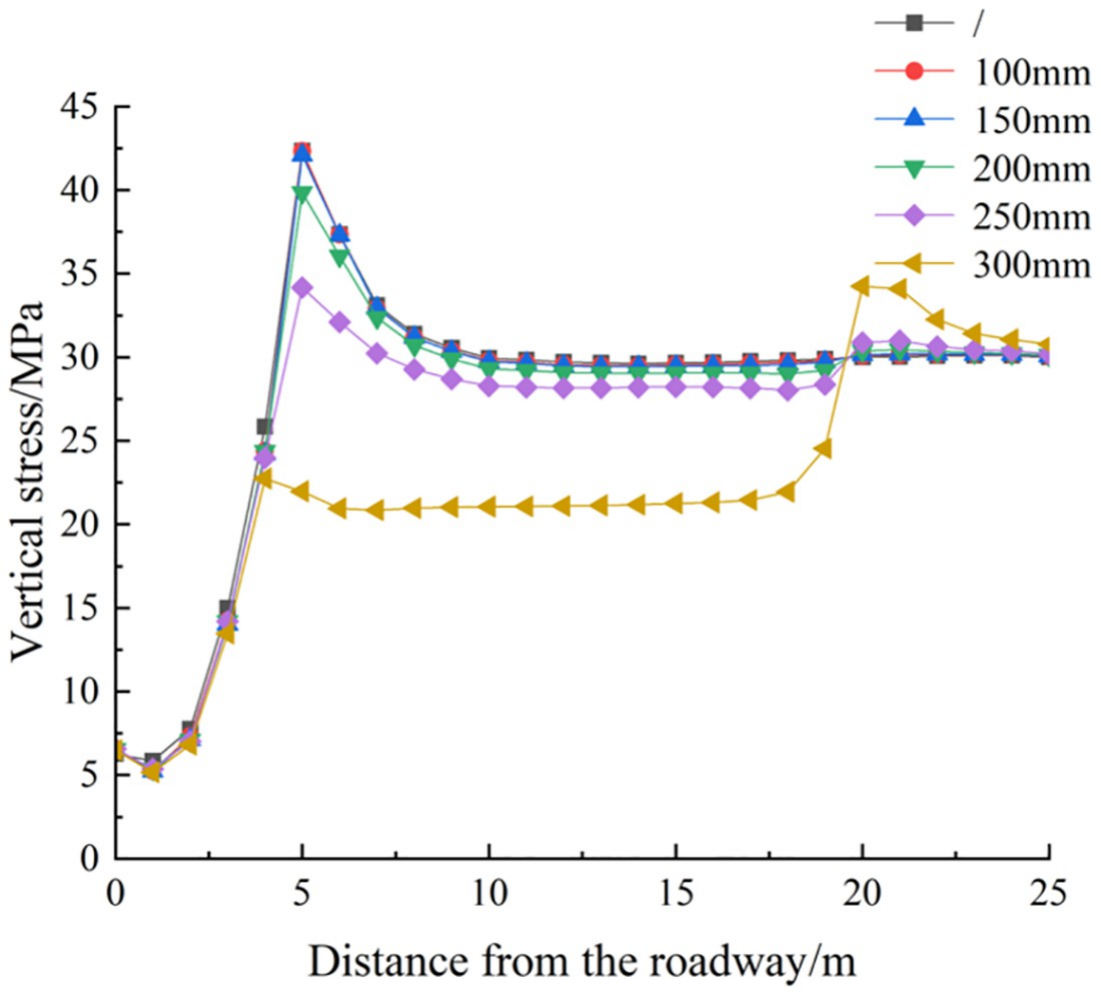
Vertical stress distribution curve of roadway surrounding rock under different deep reaming diameter.

*(1) The influence of deep reaming diameter on the vertical stress distribution of roadway side*. Fig 5 illustrates the vertical stress distribution cloud diagram of the roadway’s surrounding rock under various deep reaming diameters, subjected to both dynamic and static loads. Meanwhile, Fig 6 displays the vertical stress curve of the roadway under different deep reaming diameters. Without depressurization through borehole, the vertical stress peak of the roadway’s surrounding rock side reaches 42.65 MPa, positioned 5.0 m away from the roadway side, indicating a significant stress concentration area. With deep reaming diameters of 100 mm, 150 mm, 200 mm, and 250 mm, the vertical stress peaks of the roadway reduce to 41.14 MPa, 40.87 MPa, 39.52 MPa, and 35.05 MPa, respectively. Correspondingly, the vertical stresses at the original peak positions decrease to 40.82 MPa, 40.35 MPa, 39.21 MPa, and 34.44 MPa, reflecting reductions of 4.29%, 5.39%, 8.07%, and 19.25% compared to non-drilled conditions. Notably, the high-stress area on the roadway’s left side gradually shifts towards the deep reaming’s end, demonstrating the reaming’s effective absorption and dissipation of dynamic load stress waves. For a deep reaming diameter of 300 mm, the vertical stress peak at the roadway side decreases to 43.63 MPa, with the vertical stress at the original peak position plunging to 23.06 MPa, marking a 45.93% reduction compared to undrilled conditions. Here, the high-stress area on the roadway’s left side completely transfers to the deep reaming’s end, showcasing the most pronounced absorption and dissipation effect on dynamic stress waves.

Different parameters of deep reaming diameter exhibit varying degrees of response to dynamic loads. When the deep reaming diameter ranges from 100 mm to 200 mm, the stress distribution on the left side of the roadway undergoes minimal change compared to static conditions, resulting in a minor dynamic load response. However, with a deep reaming diameter of 300 mm, significant alterations occur in the stress distribution on the left side of the roadway, leading to a notable increase in dynamic load response. Notably, the high-stress area on the left side of the roadway relocates to the end and lower part of the deep reaming. This phenomenon primarily stems from the release of stress in the upper surrounding rock post-reaming, resulting in loosening of the rock and soil mass. Consequently, the absorption and dissipation of dynamic load stress waves are enhanced, thereby mitigating the impact of dynamic loads on the roadway.

**Fig 7:**
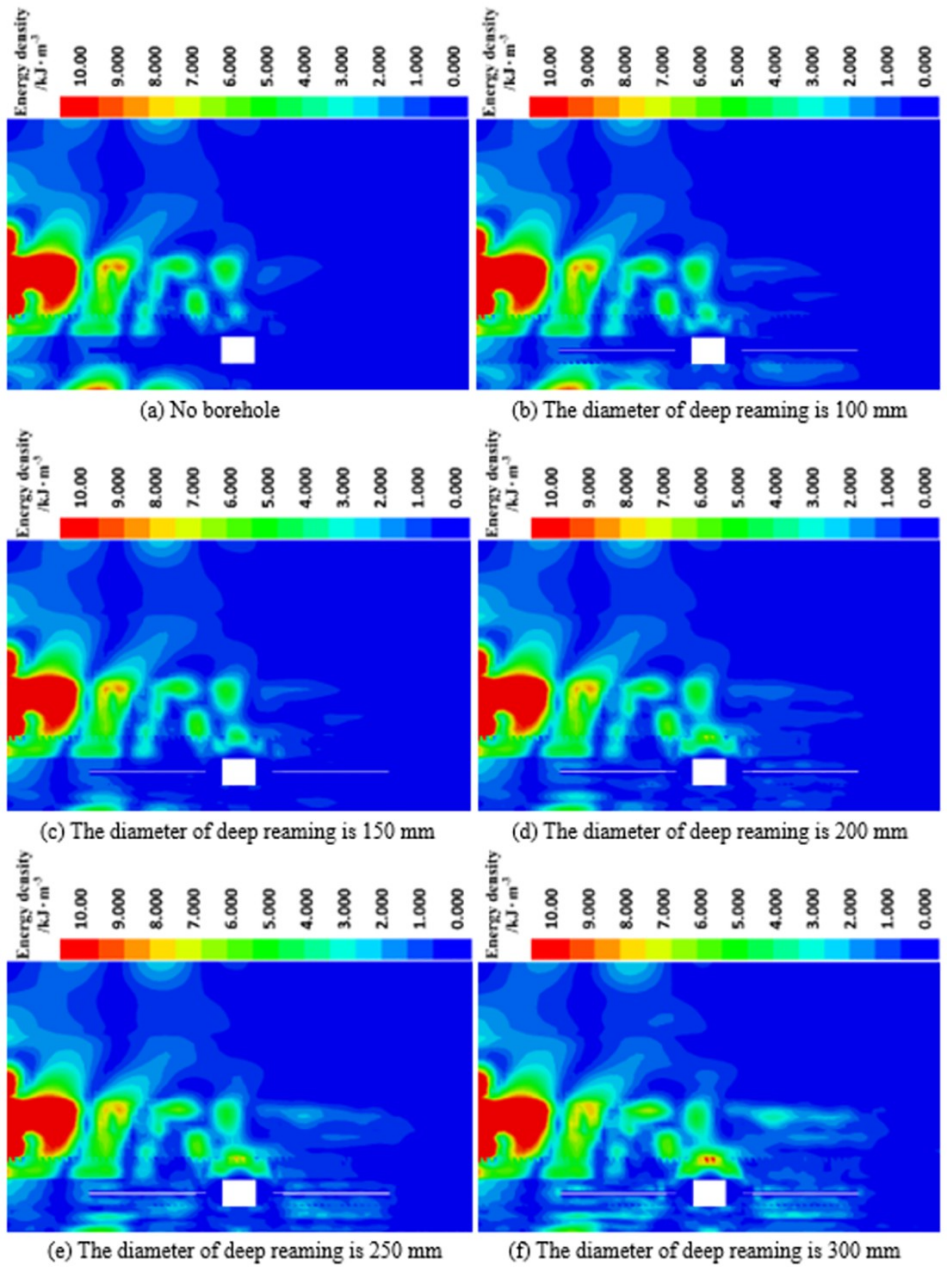
Dissipative energy density cloud diagram of roadway surrounding rock under different deep reaming diameters.

*(2) The influence of deep reaming diameter on energy dissipation*. Fig 7 depicts the dissipated energy density distribution of roadway surrounding rock under varying deep reaming diameters. A dynamic stress wave is introduced into the model from the left boundary and is promptly absorbed and dissipated upon contact with the rock and soil, leading to a notable concentration of dissipated energy density at the model’s boundary. However, due to the substantial energy dissipation at the boundary, the energy dissipation by the borehole relative to the boundary is minimal, resulting in the absence of a color band around the borehole in the model’s concentrated dissipated energy density area. To facilitate the analysis of dynamic stress wave absorption and dissipation by boreholes, adjustments were made to the color band display area, reducing the maximum value of the color band.

Observing the diagram reveals that within the same dissipation energy density range, there is minimal dissipated energy density color band on the right side of the roadway in the absence of borehole. However, as the diameter of deep reaming increases from 100 mm to 300 mm, the area of dissipated energy density around the borehole gradually expands. Particularly noteworthy is the significant concentration of dissipated energy density above the borehole on the right side of the roadway when the deep reaming diameter ranges from 250 mm to 300 mm. This observation underscores the increasingly significant effect of borehole on the absorption and dissipation of dynamic load stress waves with an increasing diameter of deep reaming.

**Fig 8:**
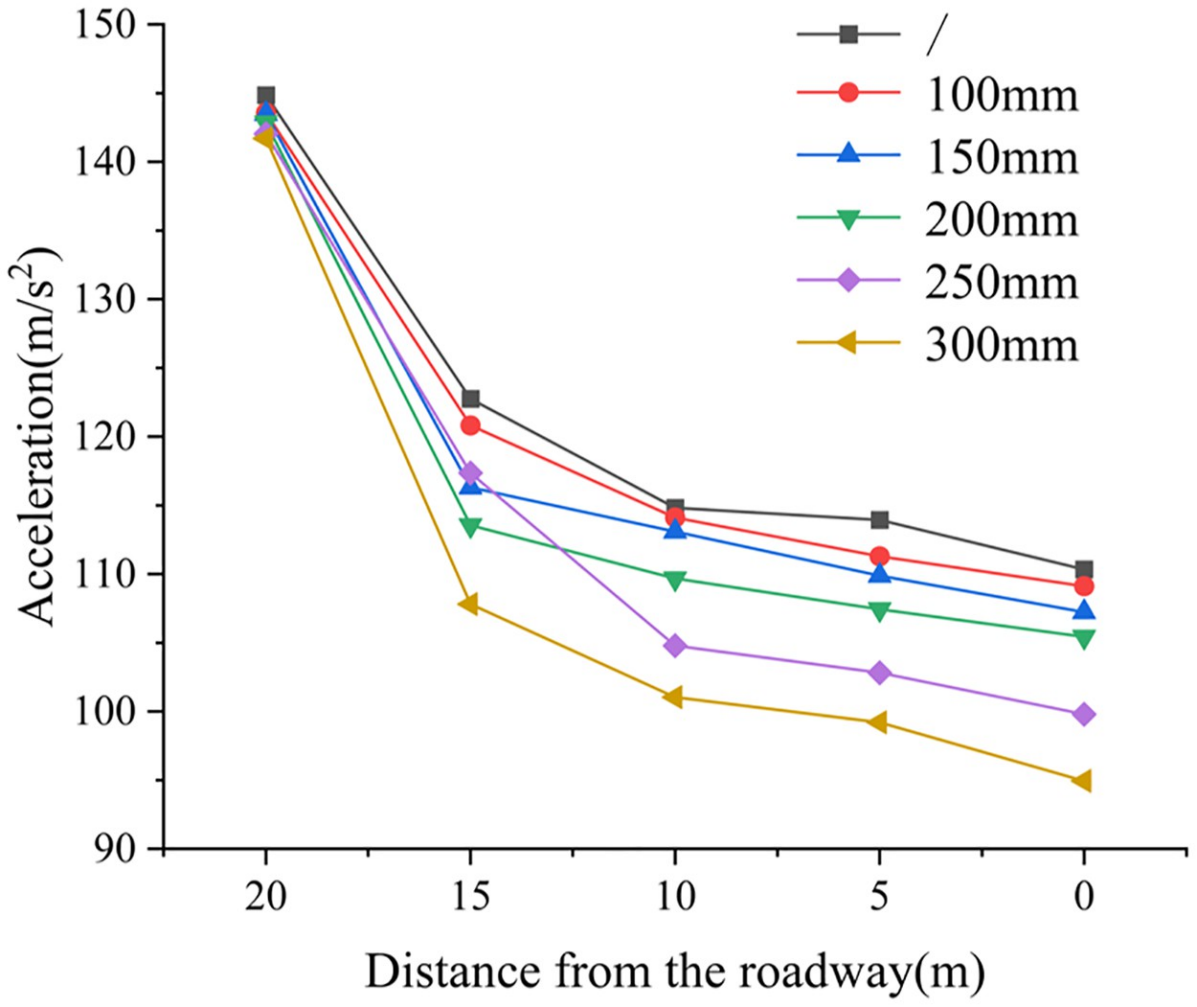
The change diagram of dynamic load acceleration peak under different deep reaming diameters.

*(3) The influence law of deep reaming diameter on dynamic load acceleration transfer*. Fig 8 illustrates the change in peak acceleration under varying deep reaming diameters. In the absence of borehole, the acceleration value 10 m away from the roadway side is 144.851 m/s^2^, while at the roadway side (0 m away), it is 110.346 m/s^2^, representing a decrease of 23.82%. For deep reaming diameters of 100 mm, 150 mm, and 200 mm, the acceleration values at the 10 m position from the roadway are 143.569 m/s^2^, 143.48 m/s^2^, and 142.86 m/s^2^, respectively. Corresponding acceleration values at the roadway side (0 m away) are 109.11 m/s^2^, 107.233 m/s^2^, and 105.43 m/s^2^, showing reductions of 24.00%, 25.26%, and 26.20%, respectively. With deep reaming diameters of 250 mm and 300 mm, the acceleration values at 10 m away from the roadway side are 142.043 m/s2 and 141.7 m/s^2^, respectively. At the roadway side (0 m away), these values are 101.79 m/s^2^ and 94.944 m/s^2^, resulting in reductions of 28.34% and 33.00%, respectively.

The diameter of the deep reaming ranges from 100 mm to 200 mm. As the diameter of deep reaming increases, the acceleration decrease also increases. However, compared to scenarios without borehole, the weak structure formed by borehole does not significantly affect the absorption and dissipation of dynamic load stress waves. This indicates that while the weak structure formed by the borehole contributes to absorbing and dissipating dynamic load stress waves, its impact is not prominent. When the diameter of deep reaming exceeds 250 mm, the acceleration decrease becomes notably more pronounced. Particularly, with a diameter of 300 mm, the acceleration decrease is the most substantial, reaching 33.00%. This suggests that deep large-diameter reaming expands the stress release range of surrounding rock in the roadway, creating a broader weak structure zone. Consequently, this plays a more significant role in absorbing and dissipating dynamic load stress waves, thereby mitigating the impact of dynamic load on the roadway.

#### 3.2.2 Numerical simulation scheme of different deep reaming depth

**Fig 9:**
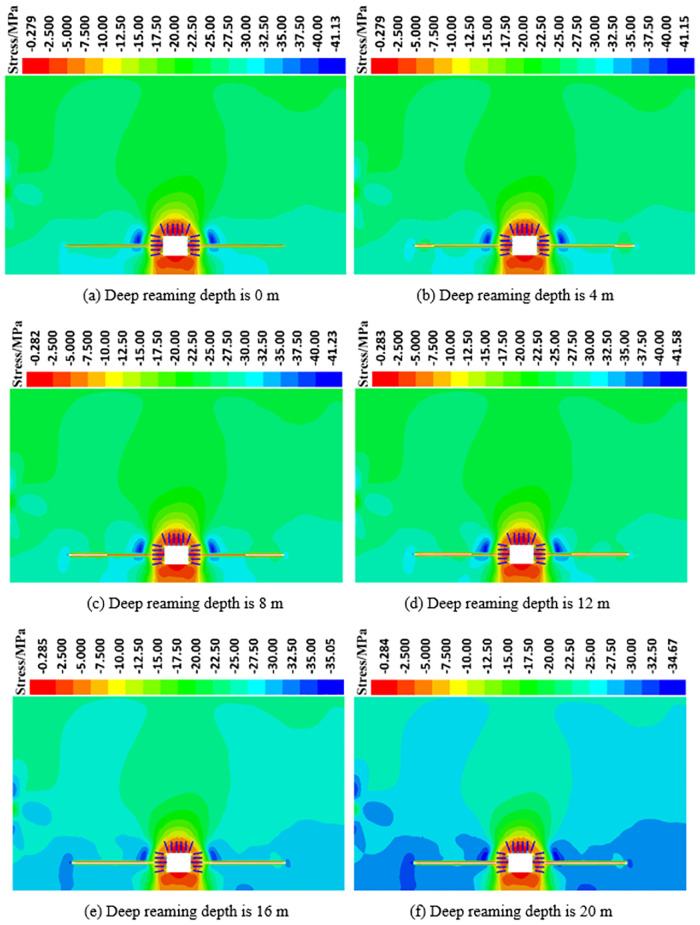
Vertical stress distribution around the roadway under different deep reaming depths.

**Fig 10:**
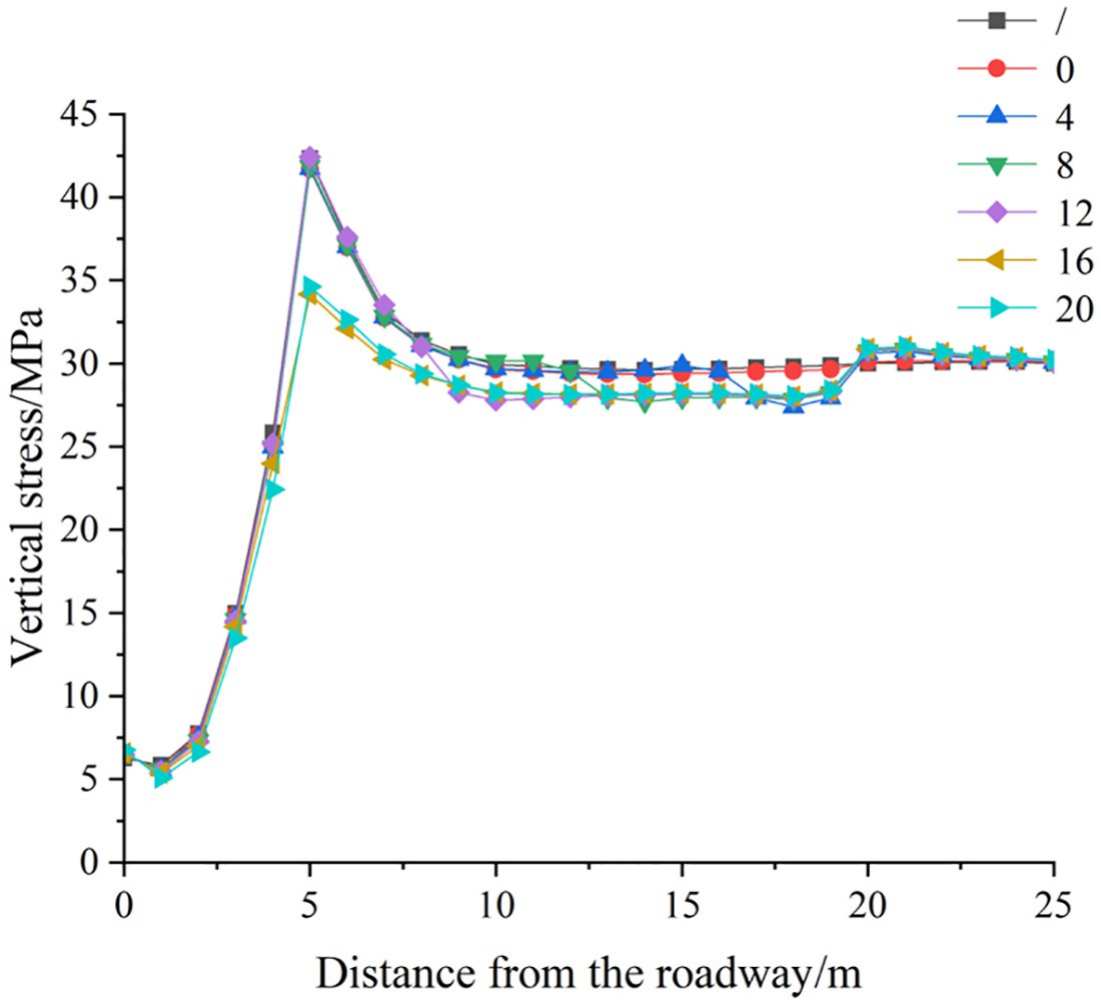
Vertical stress distribution curve of roadway surrounding rock under different deep reaming depth.

*(1) The influence of deep reaming depth on the vertical stress distribution of roadway side*. Fig 9 illustrates the vertical stress distribution cloud map of the roadway’s surrounding rock under various deep reaming depths under both dynamic and static load conditions. Fig 10 depicts the vertical stress curve of the roadway side under different deep reaming depths. In the absence of borehole, excavation of the roadway leads to the formation of a stress concentration area near the surrounding rock of the roadway side. The maximum concentration stress measures 42.65 MPa, located 5.0 m from the roadway side. As the deep reaming depth varies (0 m, 4 m, 8 m, and 12 m, with corresponding variable diameter positions of 20 m, 16 m, 12 m, and 8 m away from the roadway side), the vertical stress peaks of the roadway side register 41.13 MPa, 41.15 MPa, 41.23 MPa, and 41.58 MPa, respectively, while the vertical stress at the original stress peak position reads 40.62 MPa, 40.95 MPa, 40.88 MPa, and 40.21 MPa, respectively. Compared to undrilled holes, reductions range from 4.76% to 5.72%. The left side of the roadway’s deep reaming end starts to exhibit a high-stress area, with reaming generally contributing to the absorption and dissipation of dynamic stress waves. For deep reaming depths of 16 m and 20 m, with variable diameter positions positioned before the peak position of the roadway’s vertical stress without pressure relief (5.0 m from the roadway side), the peak vertical stress on the roadway side measures 35.05 MPa and 34.67 MPa, respectively. The original stress peak position reads 34.58 MPa and 34.46 MPa, marking reductions of 18.92% and 19.20% compared to undrilled holes. The high-stress zone on the roadway’s side extends deeper, with the high-stress zone at the end of the deep reaming on the left side of the roadway being wider and more severe. The absorption and dissipation effect of reaming on dynamic load stress waves is noticeable.

Different deep reaming depth parameters elicit varying degrees of dynamic load response. When the deep reaming depth ranges from 0 m to 12 m, the stress distribution on the left side of the roadway does not significantly differ from that without dynamic load, resulting in a small dynamic load response. However, as the deep reaming depth increases to 16 m to 20 m, there is a notable change in stress distribution, accompanied by a substantial response to dynamic load. The high-stress area on the left side of the roadway shifts towards the end of the deep reaming. This shift occurs because the stress in the upper surrounding rock is released after reaming, causing the rock and soil to loosen, which in turn absorbs and dissipates dynamic load stress waves, thereby reducing their impact on the roadway. To optimize the pressure relief effect, it is recommended that the variable diameter position be situated within the peak position of the roadway surrounding rock’s vertical stress without pressure relief, specifically within 5.0 m from the roadway side.

**Fig 11:**
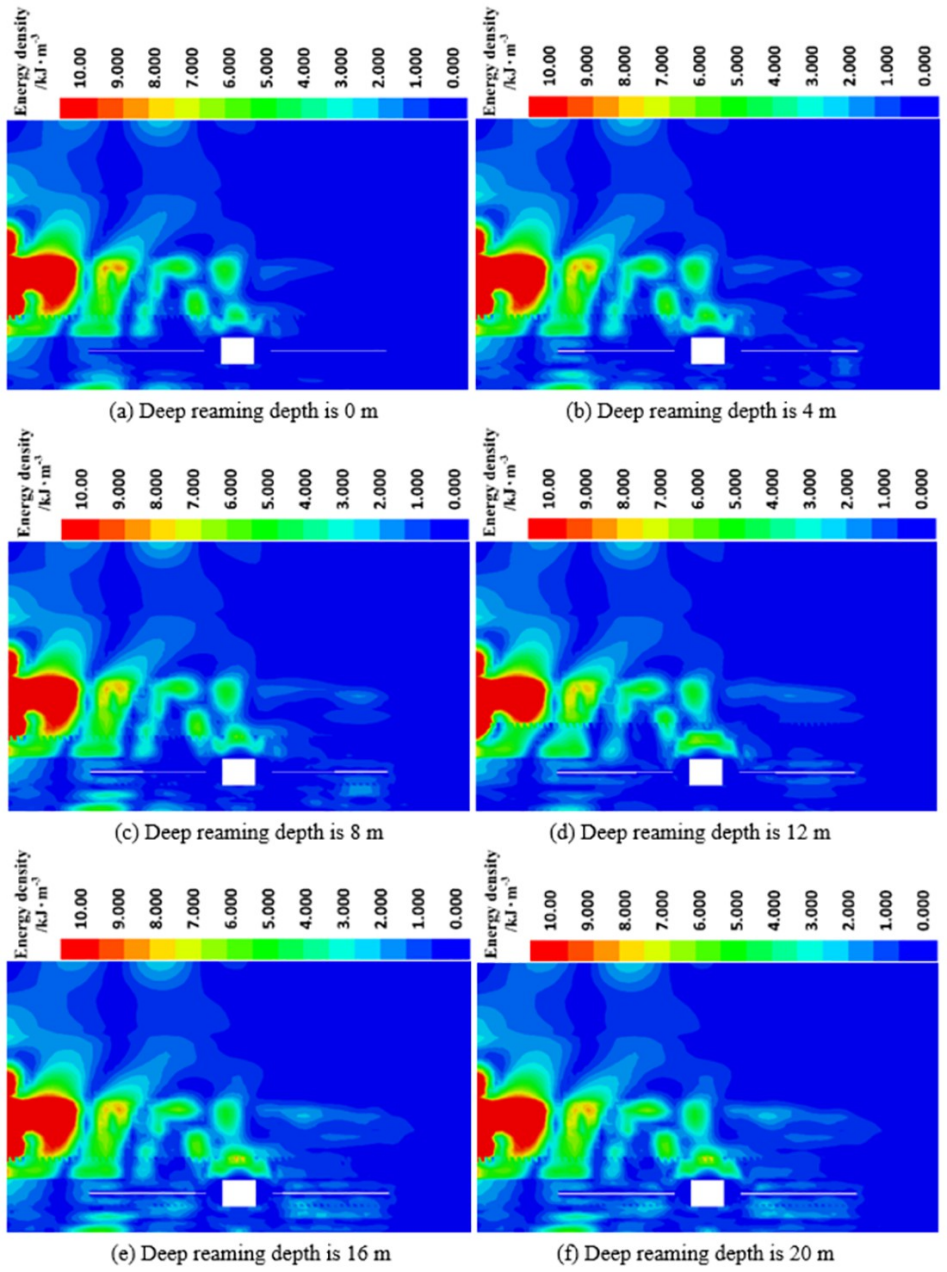
Dissipative energy density cloud diagram of roadway surrounding rock under different deep reaming depths.

*(2) The influence of deep reaming depth on energy dissipation*. Through Fig 11, it is evident that within the same dissipative energy density range, when the depth of deep reaming is 0 m (indicating no reaming in the deep), there is virtually no dissipative energy density color band on the right side of the roadway. However, as the depth of deep reaming increases from 4 m to 20 m, the area of dissipated energy density around the deep reaming gradually expands. Additionally, a substantial concentration of dissipated energy density gradually emerges above the borehole on the right side of the roadway. This observation indicates that the absorption and dissipation effect of borehole on dynamic stress waves becomes more pronounced with increasing depth of deep reaming.

**Fig 12:**
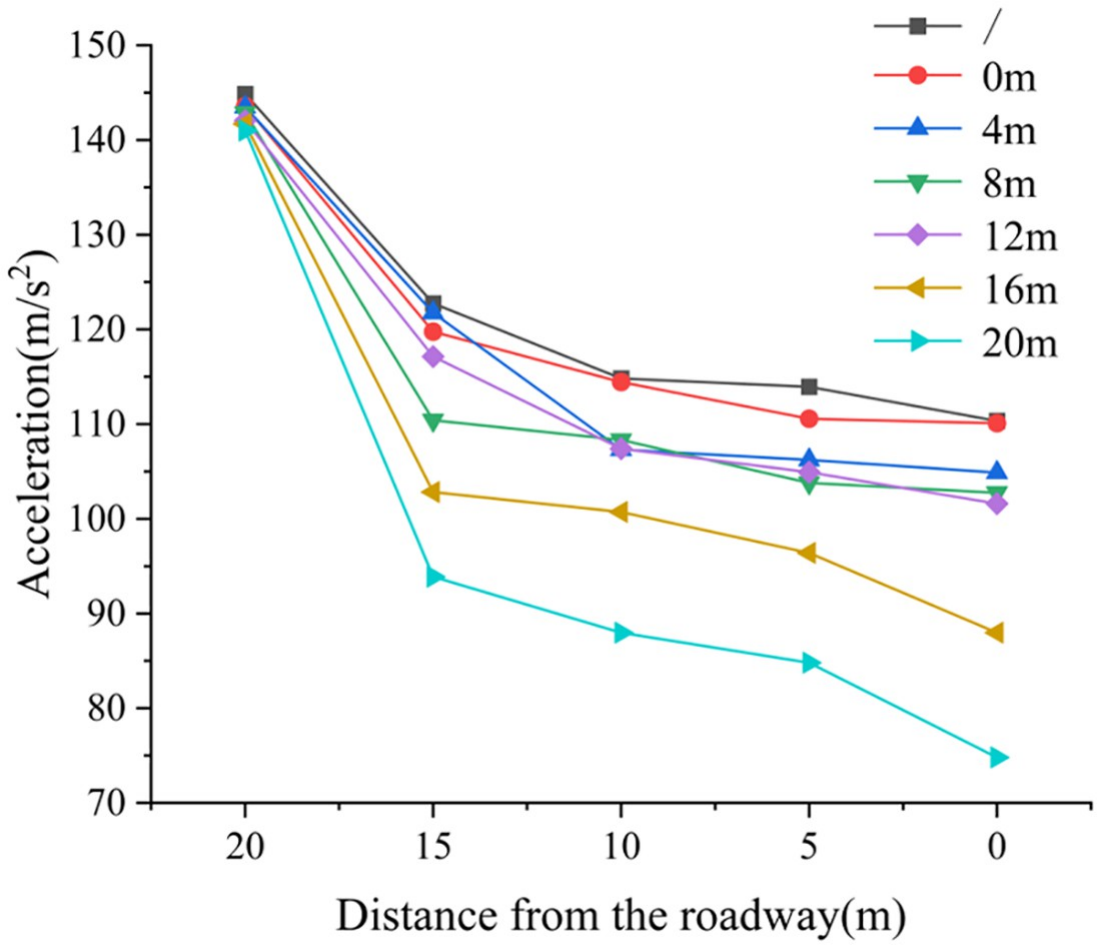
The change diagram of dynamic load acceleration peak under different deep reaming depth.

*(3) The influence law of deep reaming depth on dynamic load acceleration transfer*. Fig 12 depicts the peak acceleration change diagram of dynamic load under varying deep reaming depths. Under the condition of no borehole, the acceleration value at a distance of 10 m away from the roadway side is 144.851 m/s^2^, and at 0 m away from the roadway side, it is 110.346 m/s^2^, representing a decrease of 23.82%. When the depth of deep reaming is 0 m, 4 m, 8 m, and 12 m, the acceleration values at a distance of 10 m from the roadway are 143.569 m/s^2^, 143.48 m/s^2^, 142.86 m/s^2^, and 142.043 m/s^2^, respectively. The corresponding acceleration values at 0 m from the roadway are 110.10 m/s^2^, 104.876 m/s^2^, 102.759 m/s^2^, and 101.604 m/s^2^, showing decreases of 23.31%, 26.91%, 28.07%, and 28.47%, respectively. Moreover, when the deep reaming depth is 16 m and 20 m, the acceleration values at a distance of 10 m from the roadway are 141.7 m/s^2^ and 141.0 m/s^2^, while at 0 m from the roadway, they are 87.9717 m/s^2^ and 74.8016 m/s^2^, respectively, reflecting decreases of 37.92% and 46.95%.

The depth of deep reaming ranges from 0 m to 12 m. As the depth of deep reaming increases, the decrease in acceleration exhibits a rising trend. Comparatively, the increase in acceleration is not significant when compared to scenarios without borehole, suggesting that the weak structure formed by borehole contributes to absorbing and dissipating dynamic load stress waves, albeit with limited effectiveness, resulting in a minor degree of dynamic load response. However, when the depth of deep reaming exceeds 16 m, the acceleration decrease becomes notably pronounced. Particularly, at a depth of 20 m, the acceleration decrease is the most substantial, reaching 46.95%. This indicates that deeper deep reaming induces a broader range of stress release in the surrounding rock of the roadway side, forming a more extensive weak structure. This structure exhibits enhanced absorption and dissipation capabilities for dynamic load stress waves, resulting in a heightened dynamic load response and reduced impact on the roadway. Taking into account the influence of deep reaming depth on dynamic load response, it is recommended that the variable diameter position be located within the vertical stress peak position of roadway surrounding rock without pressure relief, specifically within 5.0 m from the roadway side.

#### 3.2.3 Numerical simulation scheme of different deep reaming spacing

**Fig 13:**
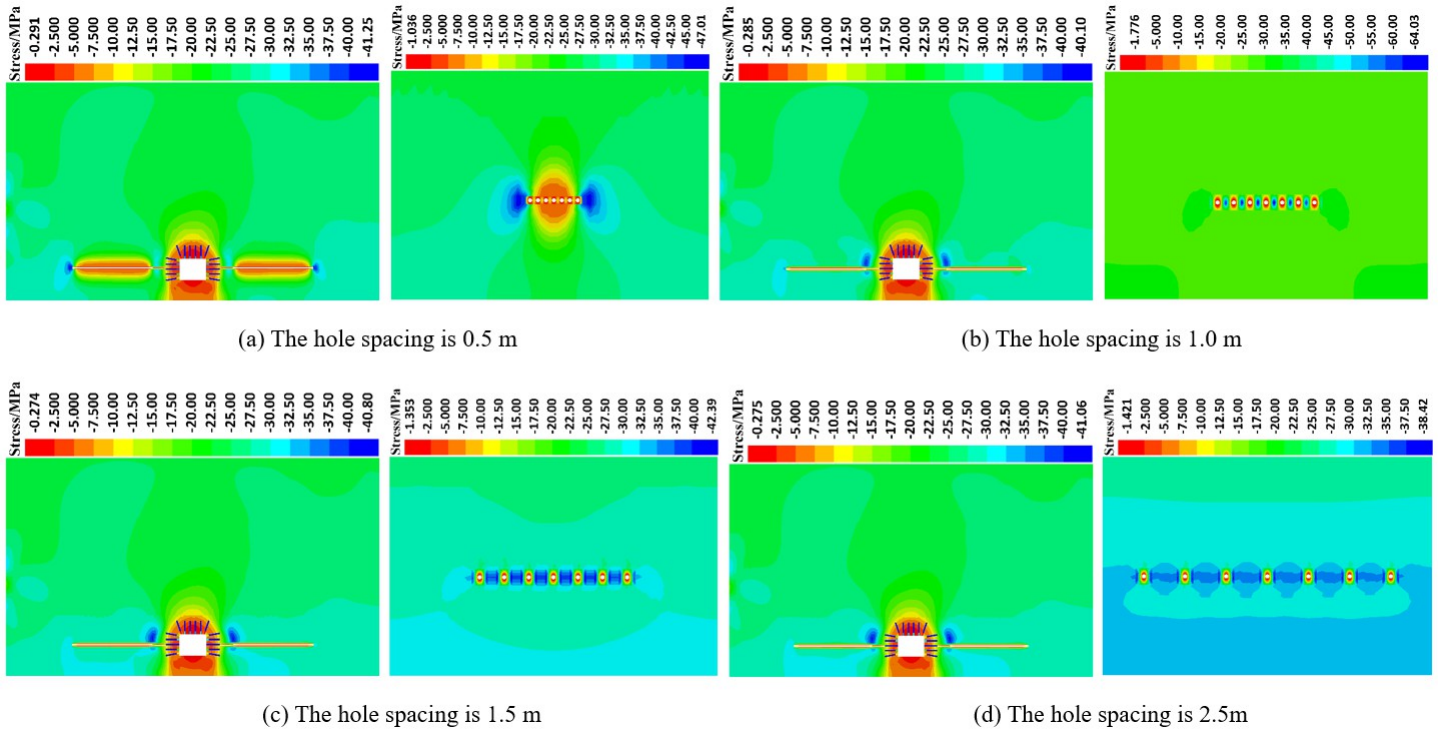
Vertical stress distribution of roadway surrounding rock under different deep reaming spacing.

**Fig 14:**
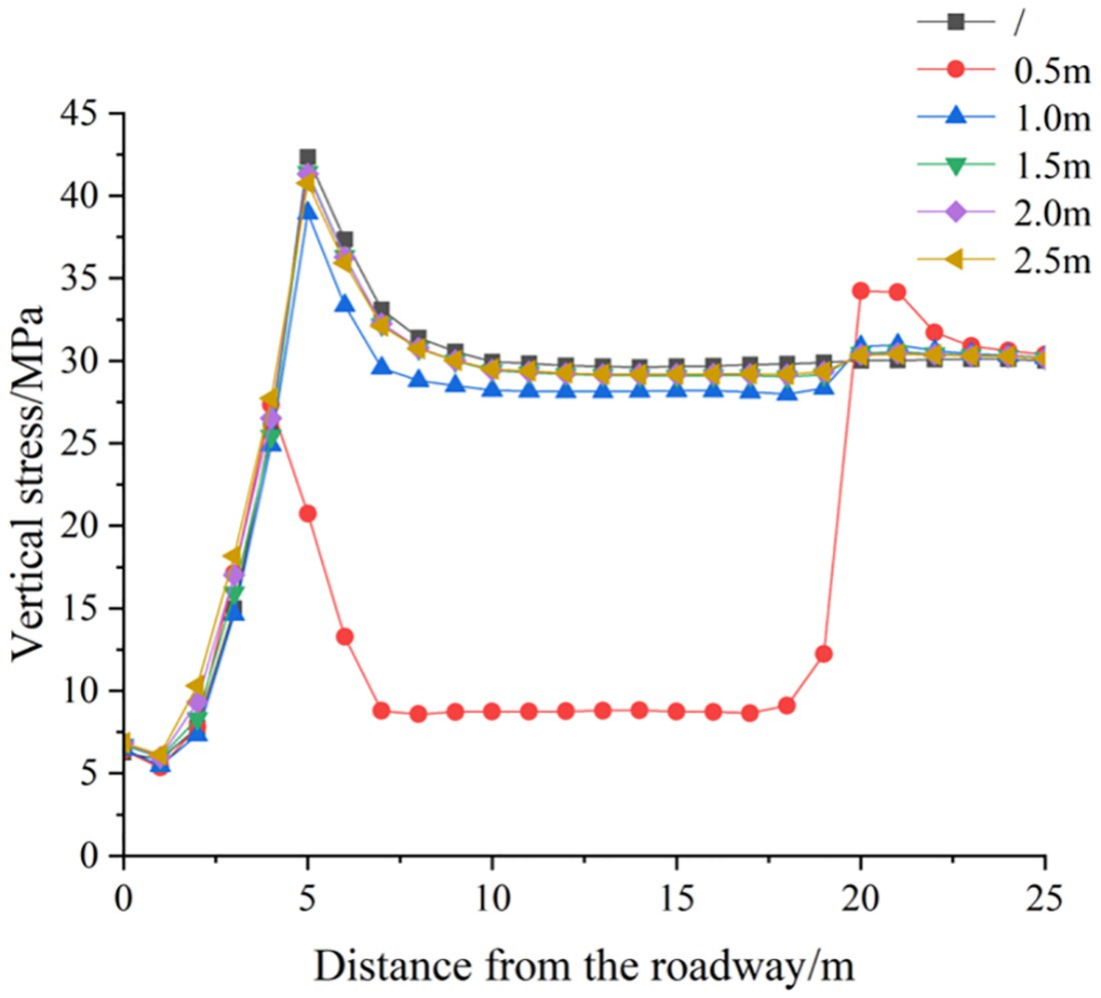
Vertical stress distribution curve of roadway surrounding rock under different deep expansion spacing.

*(1) The influence of deep reaming spacing on the vertical stress distribution of roadway side*. Fig 13 depicts the vertical stress distribution cloud diagram of the surrounding rock of the roadway under various deep reaming spacing conditions, both dynamic and static. Additionally, Fig 14 illustrates the vertical stress curve of the roadway under different deep reaming spacing. In the absence of borehole, excavation of the roadway leads to a stress concentration area near the surrounding rock of the roadway side, with the maximum stress concentration reaching 42.65 MPa, positioned 5.0 m away from the roadway side. When the borehole spacing is set to 2.5 m, 2.0 m, and 1.5 m, the vertical stress peak of the roadway measures 41.06 MPa, 40.99 MPa, and 40.80 MPa, respectively. Correspondingly, the stress at the original peak position is 39.91 MPa, 39.87 MPa, and 39.76 MPa, representing reductions of 6.42%, 6.52%, and 6.78% compared to undrilled conditions. Notably, a high-stress zone forms at the bottom and end of the deep reaming, with the reaming exhibiting a general effect on absorbing and dissipating dynamic load stress waves. When the borehole spacing is reduced to 1.0 m, the vertical stress peak of the roadway side measures 40.10 MPa, while the stress at the original peak position is 39.84 MPa, representing a 6.59% reduction compared to the scenario without borehole pressure relief. Furthermore, the stress concentration at the end of the deep reaming on the left side of the roadway intensifies, indicating improved absorption and dissipation effects of the reaming on dynamic load stress waves. With a borehole spacing of 0.5 m, the vertical stress peak of the roadway side measures 41.25 MPa, while the stress at the original peak position is 25.11 MPa, marking a substantial 41.13% reduction compared to undrilled conditions. Here, the high-stress area of the roadway side is entirely transferred to the end of the deep reaming, with a stress concentration area emerging at the end of the deep reaming. Consequently, the reaming demonstrates a significant impact on the absorption and dissipation of dynamic stress waves.

Different borehole spacing parameters elicit varying degrees of dynamic load response. When the borehole spacing is set to 2.5 m, 2.0 m, and 1.5 m, the stress distribution on the left side of the roadway due to deep reaming shows negligible deviation from conditions without dynamic load, resulting in a minimal dynamic load response. However, with borehole spacing reduced to 1.0 m and 0.5 m, significant changes occur in the stress distribution on the left side of the roadway, leading to a heightened dynamic load response. Consequently, the high-stress area shifts to the end of the deep reaming. This phenomenon can be attributed to the denser borehole pattern, resulting in a fully connected pressure relief area with the low-stress region, thereby enhancing the pressure relief effect of shallow borehole throughout the entire length of the deep reaming. Moreover, post-reaming, the stress in the upper surrounding rock dissipates, causing the rock and soil to loosen, thereby absorbing and dissipating dynamic load stress wave transmission and further mitigating the impact of dynamic load on the roadway.

**Fig 15:**
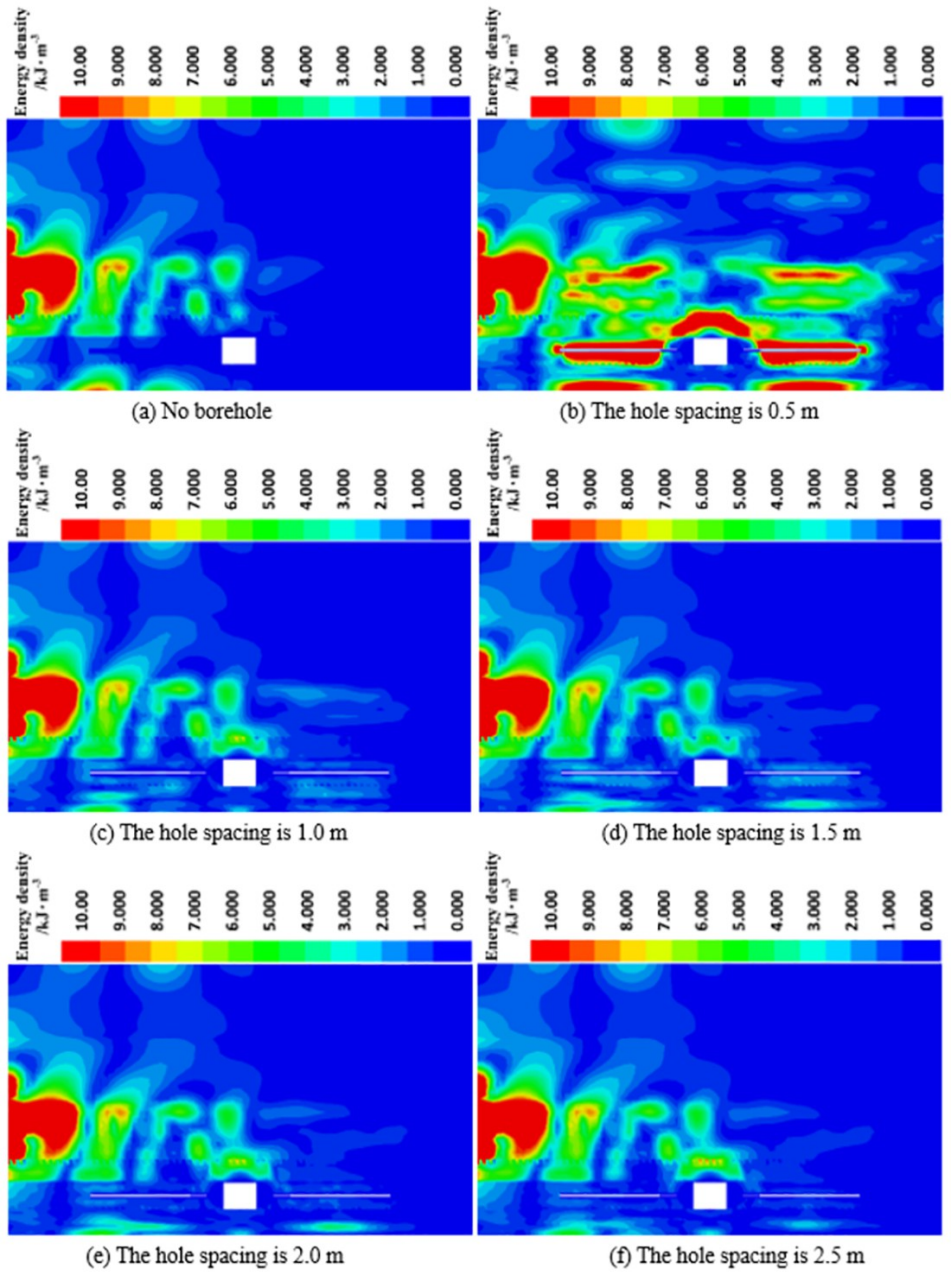
Dissipative energy cloud diagram of roadway surrounding rock under different deep reaming spacing.

*(2) The influence of deep reaming spacing on energy dissipation*. It can be observed from Fig 15 that within the same dissipation energy density range, as the deep reaming spacing gradually decreases from 2.5 m to 0.5 m, the area of dissipation energy density around the deep reaming progressively expands. This expansion is attributed to the increasing density of boreholes within a specific volume, resulting in a stronger pressure relief effect. Particularly, when the deep reaming spacing is reduced to 0.5 m, extensive areas of concentrated dissipative energy density emerge around the deep reaming. This observation underscores the heightened absorption and dissipation effect of borehole on dynamic load stress waves with increasing depth of deep reaming. Notably, at a spacing of 0.5 m, where the number of boreholes within a specific volume is the highest, the pressure relief amplitude is maximal, thus yielding optimal absorption and dissipation of dynamic load stress waves.

**Fig 16:**
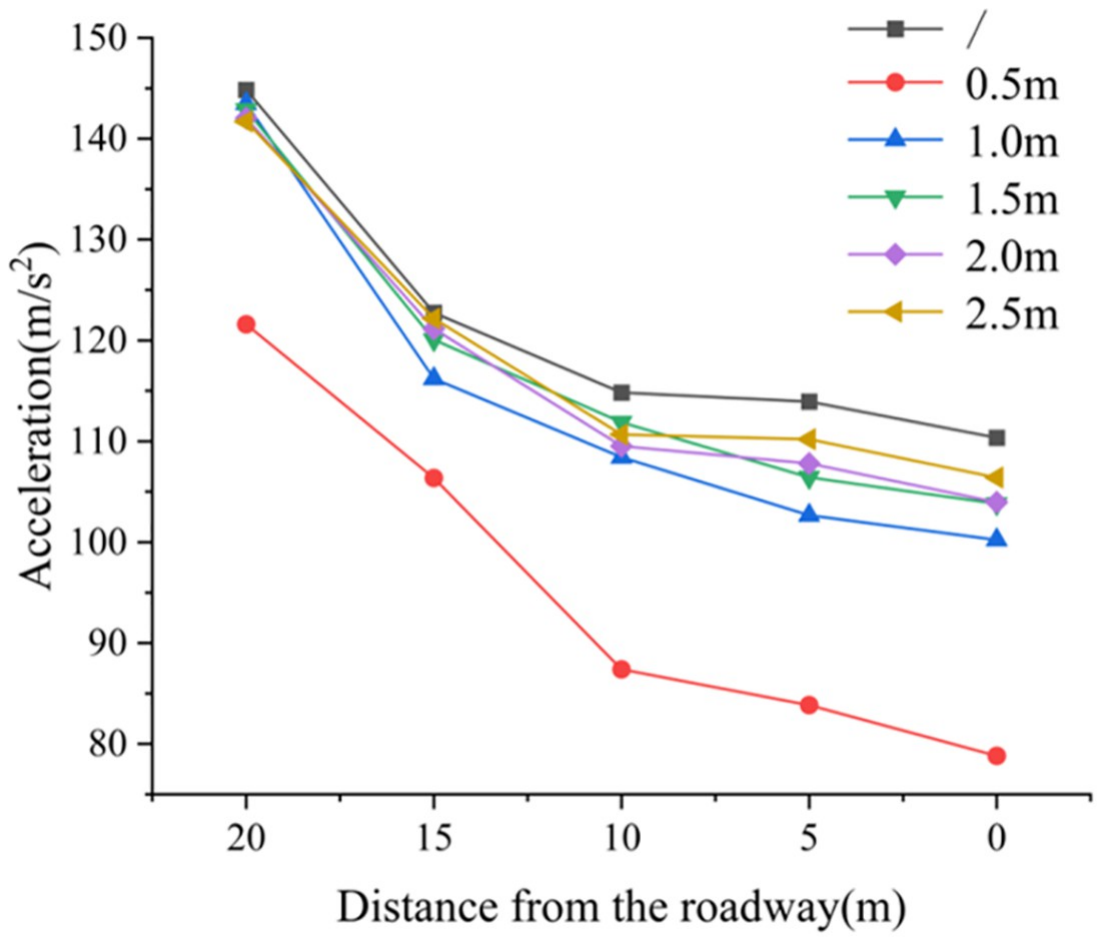
Variation of peak dynamic load acceleration at deep reaming spacing.

*(3) The influence law of deep reaming spacing on dynamic load acceleration transmission*. Fig 16 illustrates the peak acceleration change diagram of dynamic load under varying deep reaming depths. In the absence of borehole, the acceleration at 10 m away from the roadway side measures 144.851 m/s^2^, while at the roadway side (0 m distance), it is 110.346 m/s^2^, resulting in a decrease of 23.82%. With deep reaming spacings of 1.0 m, 1.5 m, 2.0 m, and 2.5 m, the acceleration values at 10 m from the roadway side are 143.48 m/s^2^, 142.86 m/s^2^, 142.043 m/s^2^, and 141.7 m/s^2^, respectively. Correspondingly, at the roadway side, the acceleration values are 100.224 m/s^2^, 103.8194 m/s^2^, 103.9731 m/s^2^, and 106.419 m/s^2^, marking decreases of 30.15%, 27.33%, 26.80%, and 24.90%, respectively. For a deep reaming spacing of 0.5 m, the acceleration at 10 m from the roadway side is 121.602 m/s^2^, while at the roadway side, it is 78.8169 m/s^2^, representing a reduction of 35.18%.

The spacing of deep reaming ranges from 1.0 m to 2.5 m. As the reaming spacing decreases, the range of acceleration reduction increases. This phenomenon occurs because the borehole holes become denser within a certain volume. However, compared to scenarios without borehole, the reduction in acceleration is not significant, indicating a minor dynamic load response. This suggests that the weak structure formed by the borehole hole contributes to absorbing and dissipating the dynamic load stress wave to some extent, albeit not prominently. When the deep reaming spacing is less than 0.5 m, the acceleration reduction significantly increases, reaching 35.18%. This can be attributed to the dense borehole, which leads to the complete penetration of the pressure relief zone and the low-stress zone generated by the borehole. Consequently, a wide weak structure forms, resulting in a substantial response to dynamic load, enhancing the absorption and dissipation of dynamic load stress wave transmission, and mitigating the impact of dynamic load on the roadway.

## 4. Physical similar simulation test

### 4.1 Test scheme

**Fig 17:**
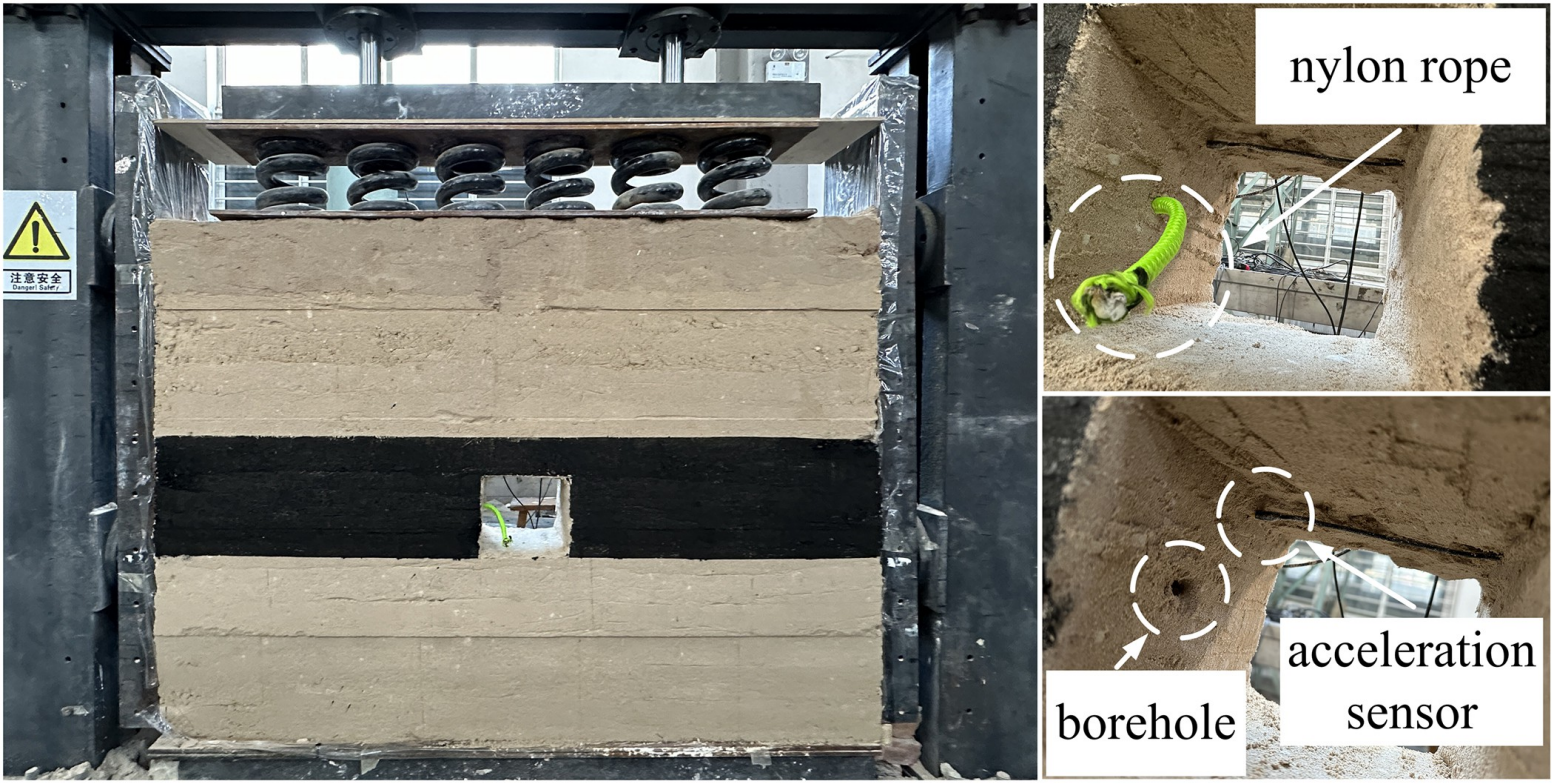
Experimental model and borehole schematic diagram.

Currently, researchers have conducted impact test studies on the surrounding rock of roadways under dynamic and static loads, primarily using the Split Hopkinson Pressure Bar (SHPB) device for simulation testing. Traditional and improved SHPB equipment is limited to small-scale test samples, making it challenging to investigate the impact process of roadway surrounding rock under combined dynamic and static conditions. Therefore, to address this limitation, we enhanced an existing laboratory simulation test device by integrating hydraulic cylinders and dumbbells. This modification enables the device to apply both static and dynamic loads, facilitating the study of the impact response of variable diameter boreholes in roadway surrounding rock under dynamic and static loading conditions. The experimental setup is depicted in Fig 17.

The test is divided into two groups: conventional borehole and variable diameter borehole. The conventional borehole has a diameter of 2 cm and a length of 40 cm. In the variable diameter borehole, the shallow part features a small diameter of 2 cm and a length of 20 cm, while the deep part includes a large diameter reaming of 8 cm with a length of 20 cm. Due to the structure of the variable diameter borehole, which is wider internally and narrower externally, excavation of the model after construction is not feasible. Consequently, a metal pipe is pre-buried, making model extraction post-drying impossible. Thus, the pre-buried wire method (using thick nylon rope) is employed to capture images from the model’s position to the borehole. In the implementation of conventional borehole, the pre-designed borehole position is laid with nylon rope. For variable diameter borehole, nylon rope is also laid in the pre-designed borehole position, with several folds made at the location of the deep large diameter borehole for easier extraction (preventing potential entanglement). Additionally, the end of the nylon rope is extended to the roadway to facilitate extraction.

The dynamic load loading device consists of a pendulum dynamic load loading apparatus. Dumbbells and thick ropes function as pendulums, falling from a height around the moving shaft, converting gravitational potential energy into impact kinetic energy. Adjusting the pendulum’s height enables obtaining different energy levels for impact dynamic load. The pendulum strikes the incident rod to transfer impact kinetic energy to the model. Following one cycle of operation, the pendulum dynamic load loading device is stopped using brakes, simulating the influence of a single dynamic load. To streamline the test model, energy attenuation during the pendulum impact’s energy transfer process is approximately disregarded.

Through the design of the pendulum dynamic loading device, the dumbbell weight is 15 kg, and the maximum drop is 1.5 meters. According to the calculation results, the maximum gravitational potential energy that can be input is 15 × 9.8 × 1.5J = 220.5J, and the actual simulated potential energy of 1.32 × 10^5^J can be obtained by energy similarity ratio conversion, which is consistent with the large energy mine earthquake energy level in the actual production of the mine.

**Fig 18:**
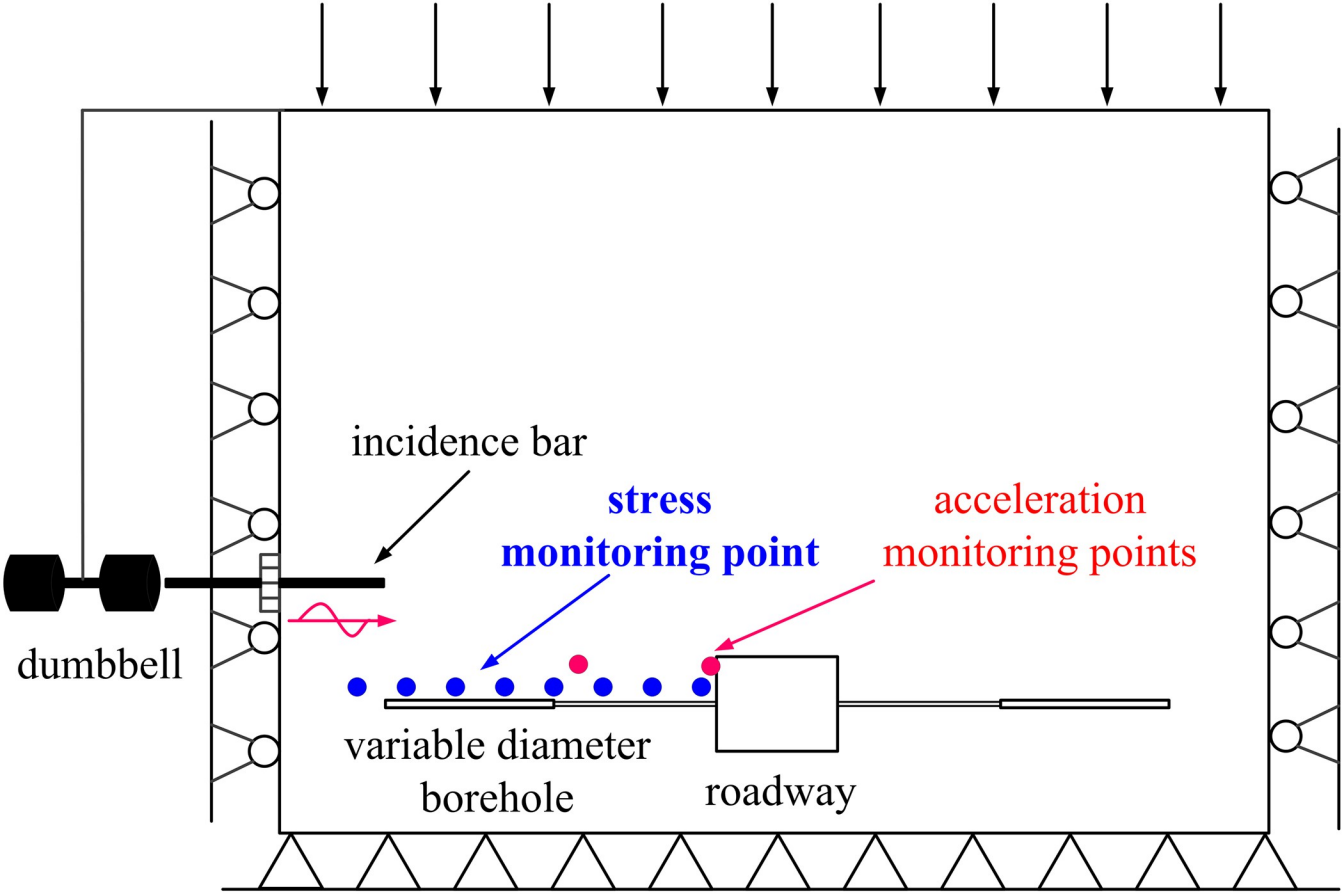
Monitoring point layout.

**Fig 19:**
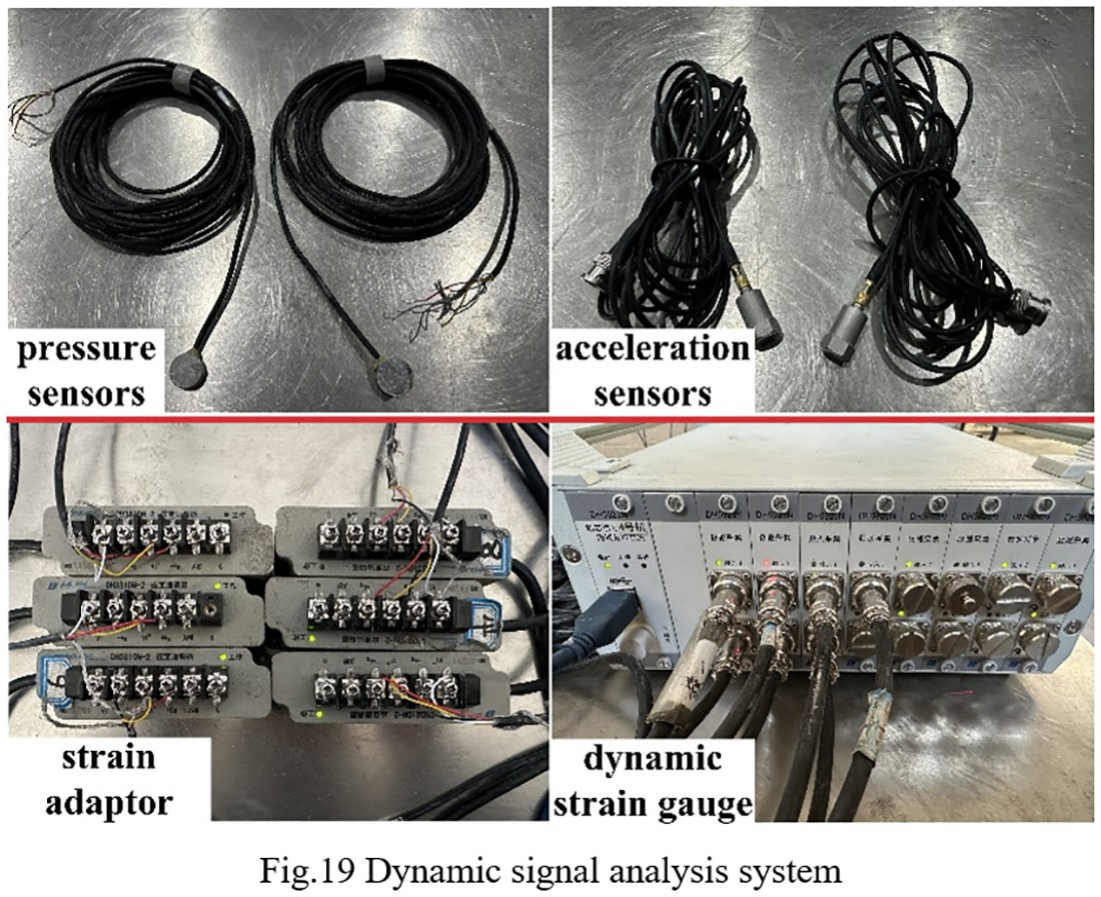
Dynamic signal analysis system.

In order to investigate the impact of conventional borehole and variable diameter borehole on the stress distribution in surrounding rock of roadways, six stress monitoring points are strategically positioned within the model. The LY-350 micro pressure sensors are utilized to record the stress variations on the roadway side at each designated point. A monitoring line, situated 250 mm above the borehole, observes stress changes in the surrounding rock following excavation. The pressure sensors are placed at a distance of 195 mm from the left boundary, with adjacent measuring points spaced 60 mm apart, as depicted in Fig 18. Data acquisition and analysis are facilitated by the DH5923N dynamic stress and strain tester, serving as the terminal for signal reception and conversion. The collected data are processed and analyzed using the DHDAS signal acquisition and analysis system on the computer side (refer to Fig 19). To explore the impact of variable diameter boreholes on the transmission characteristics of dynamic load stress waves in the surrounding rock of roadways, as well as their capacity for absorbing and dissipating dynamic load stress waves, a survey line is positioned 500 mm above the borehole. The first acceleration sensor is buried 200 mm from the roadway side to monitor the absorption and dissipation of dynamic load stress waves by the large diameter boreholes. Meanwhile, the second acceleration sensor is embedded on the left side of the roadway to monitor the transmission of dynamic load stress waves to the roadway edge. The layout arrangement is illustrated in Fig 18.

### 4.2 The stress variation law of variable diameter borehole in roadway surrounding rock

**Fig 20:**
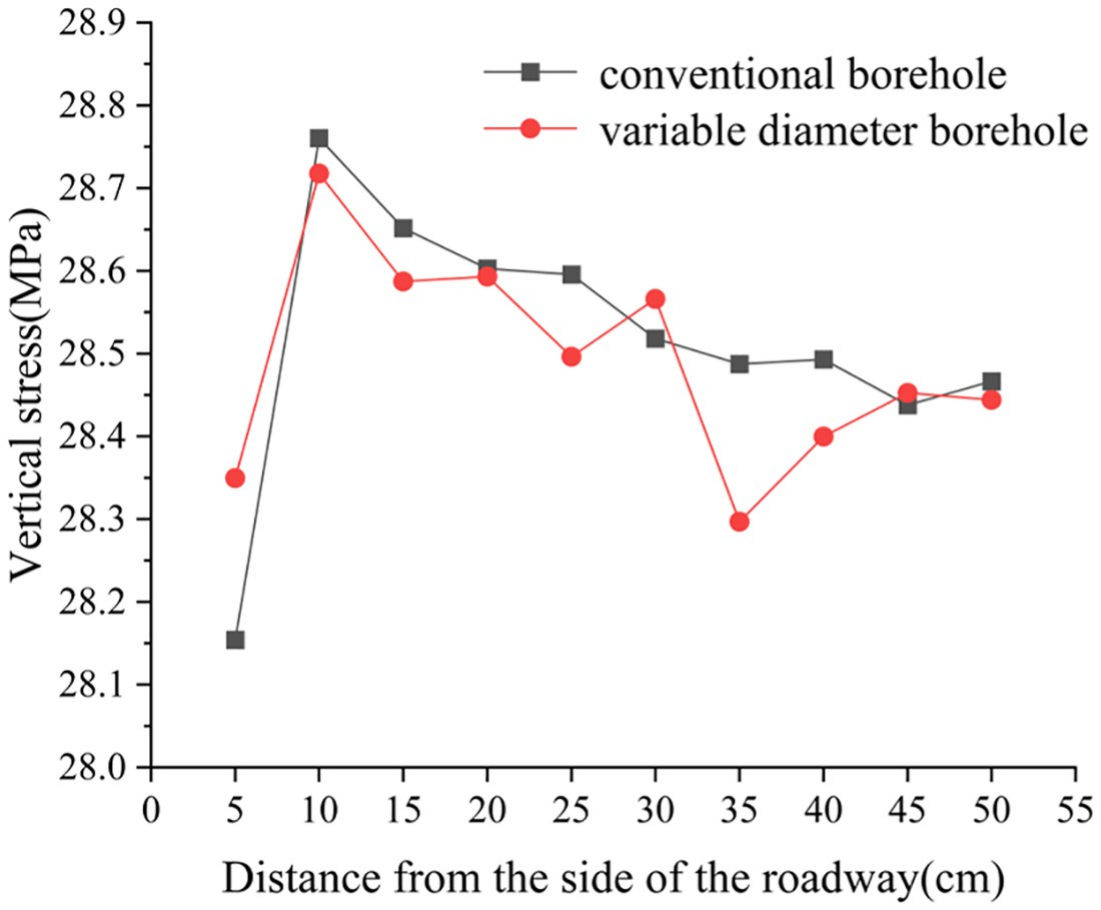
Vertical stress monitoring curve.

**Fig 21:**
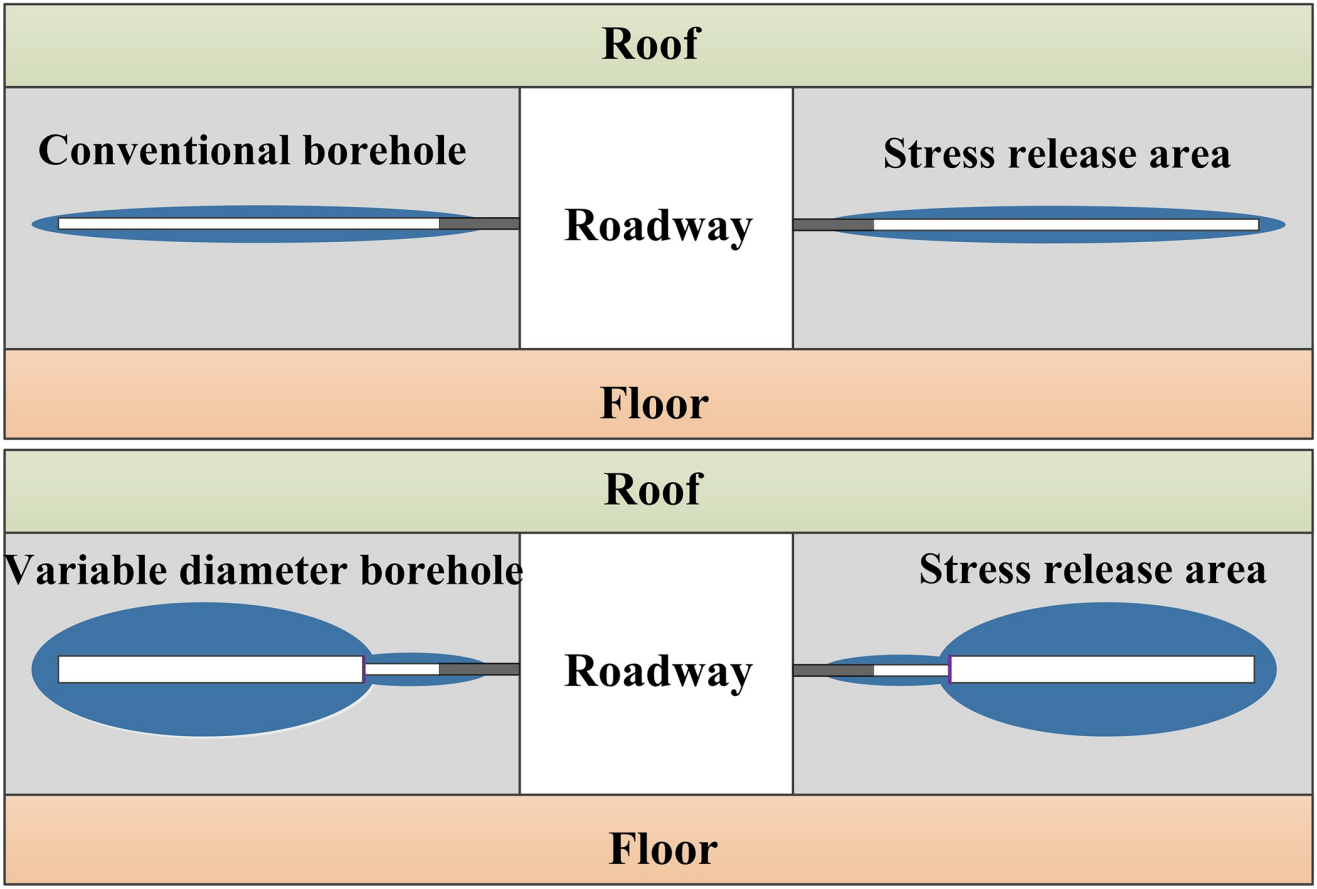
Comparison diagram of borehole stress release zone.

Fig 20 illustrates the vertical stress distribution curve of the roadway’s surrounding rock monitored in two sets of tests conducted under dynamic and static load conditions. Fig 21 is the effect diagram of stress release zone of conventional borehole and variable diameter borehole. To facilitate discussion, stress data collected from conventional borehole and variable diameter borehole tests are converted and analyzed based on the designed stress similarity ratio. Under static load conditions, vertical stress data are recorded at monitoring points prior to applying dynamic loads in similar simulation tests. The maximum vertical stress observed in both test groups originates from a monitoring point located 10 cm from the roadway side, coinciding with the stress concentration zone of the roadway’s side. Specifically, the maximum vertical stress recorded in the conventional borehole test scheme is 28.76 MPa, while the maximum stress in the variable diameter borehole test scheme is 28.72 MPa. Conversely, the minimum vertical stress at 5 cm from the roadway side reflects stress release due to roadway excavation, measuring 28.15 MPa in the conventional borehole test and 28.35 MPa in the variable diameter borehole test. Monitoring points positioned at 15 cm, 20 cm, 25 cm, 30 cm, 35 cm, and 40 cm from the roadway are situated above the borehole. The excavation process creates a cavity in the rock and soil above the borehole, leading to stress reduction after borehole and pressure relief. Consequently, vertical stress monitored at these points decreases in both conventional borehole and variable diameter borehole tests. Although both borehole methods maintain consistency within 20 cm from the roadway side, the variable diameter borehole test features large-diameter reaming between 20 cm and 40 cm from the roadway side. While ideal conditions suggest identical data for all four monitoring points within this range, the final test results indicate slightly lower vertical stress values in the variable diameter borehole test compared to the conventional borehole test. This difference could be attributed to the pressure relief range of the large-diameter borehole behind the variable-diameter borehole, or potential discrepancies in hole shapes resulting from the process of extracting the thick nylon rope, along with inherent errors in the model accumulation process.

There are significant differences between the two sets of test schemes within the 20 cm to 40 cm range from the roadway side. The conventional borehole test scheme involves small-diameter borehole in this interval, whereas the variable diameter borehole test scheme entails large-diameter reaming. The primary monitoring points in this test are the four pressure boxes located at 25 cm, 30 cm, 35 cm, and 40 cm from the roadway side. The test results reveal notable discrepancies in the values extracted from the monitoring points at 35 cm from the roadway side in the two test schemes. Specifically, the value obtained from the variable diameter borehole test scheme is 28.30 MPa, whereas the data from the conventional borehole test scheme is 28.49 MPa, representing a difference of 0.19 MPa. At distances of 25 cm, 35 cm, and 40 cm from the roadway side, the vertical stress values extracted from the monitoring points in the conventional borehole test scheme are 28.60 MPa, 28.49 MPa, and 28.49 MPa, respectively. In contrast, the vertical stress values from the monitoring points in the variable diameter borehole test scheme are 28.50 MPa, 28.30 MPa, and 28.40 MPa, respectively. Comparatively, at these four monitoring points, the variable diameter borehole test scheme exhibits a reduction in vertical stress of 0.1 MPa, 0.19 MPa, and 0.09 MPa, with reduction ranges of 0.35%, 0.67%, and 0.32%, respectively. The large-diameter reaming in the variable diameter borehole test scheme demonstrates a more effective pressure relief role. It’s worth noting that the data extracted from the monitoring point at 30 cm away from the roadway side in the variable diameter borehole test scheme is slightly larger than that in the conventional borehole test scheme, constituting an abnormal data point. This anomaly can be attributed to its location within the range of small-diameter borehole and large-diameter reaming variable aperture. The stress release of the soil layer above the large-diameter reaming can result in a small range of stress concentration on both sides, leading to a slightly higher vertical stress value extracted from this monitoring point.

### 4.3 Acceleration transfer attenuation law of variable diameter borehole in roadway surrounding rock

Dynamic loading on roadway surrounding rock induces sudden dynamic waves, resulting in substantial acceleration within the rock mass. Newton’s second law can be employed to assess the dynamic stress on roadway surrounding rock based on its acceleration response. Acceleration serves as a supplementary metric for assessing the stability of roadway surrounding rock. Excessive acceleration in roadway surrounding rock can jeopardize the stability of deeper rock layers, posing risks to both engineering structures and personnel safety. Hence, investigating the acceleration response of roadway surrounding rock under dynamic loading holds significant practical implications. Comparative analysis of acceleration variations in roadway surrounding rock under conventional and variable diameter borehole conditions aids in advancing our understanding of the progression of roadway instability and failure.

**Fig 22:**
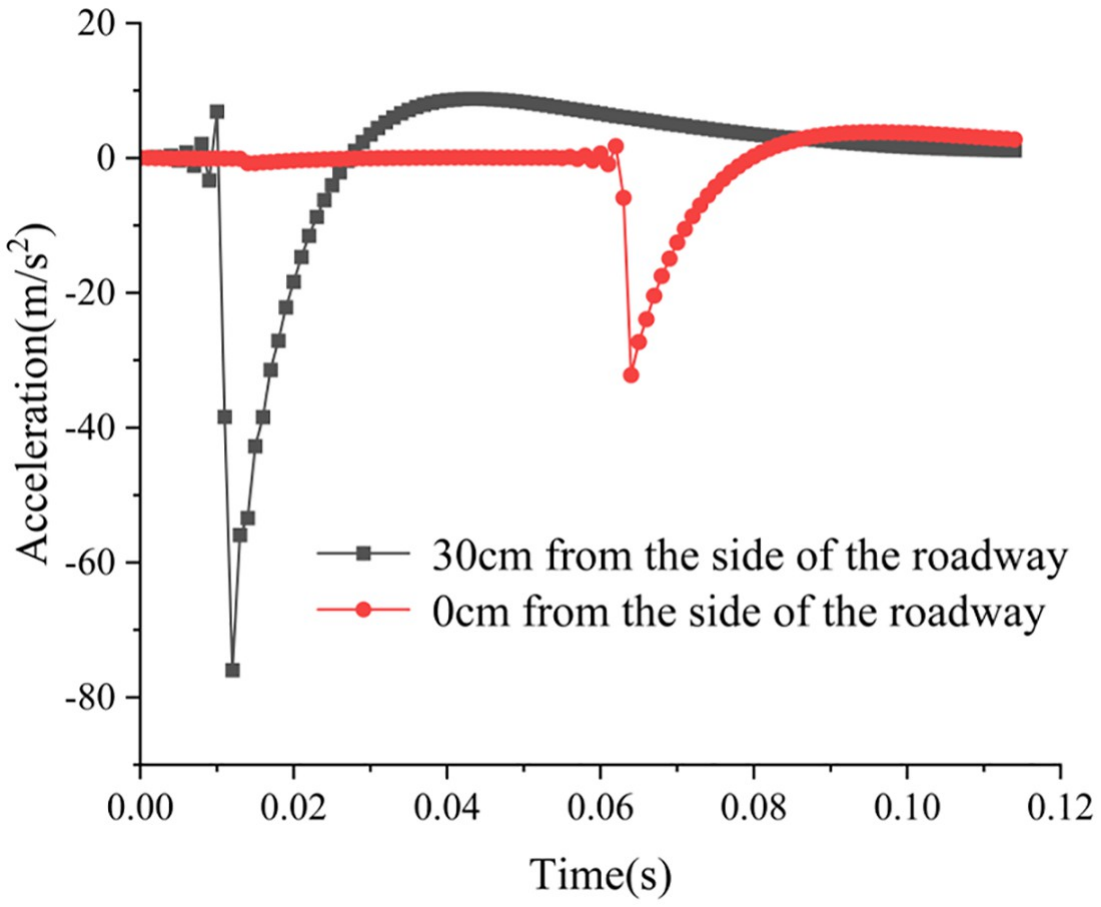
Acceleration change curve of conventional borehole test scheme.

**Fig 23:**
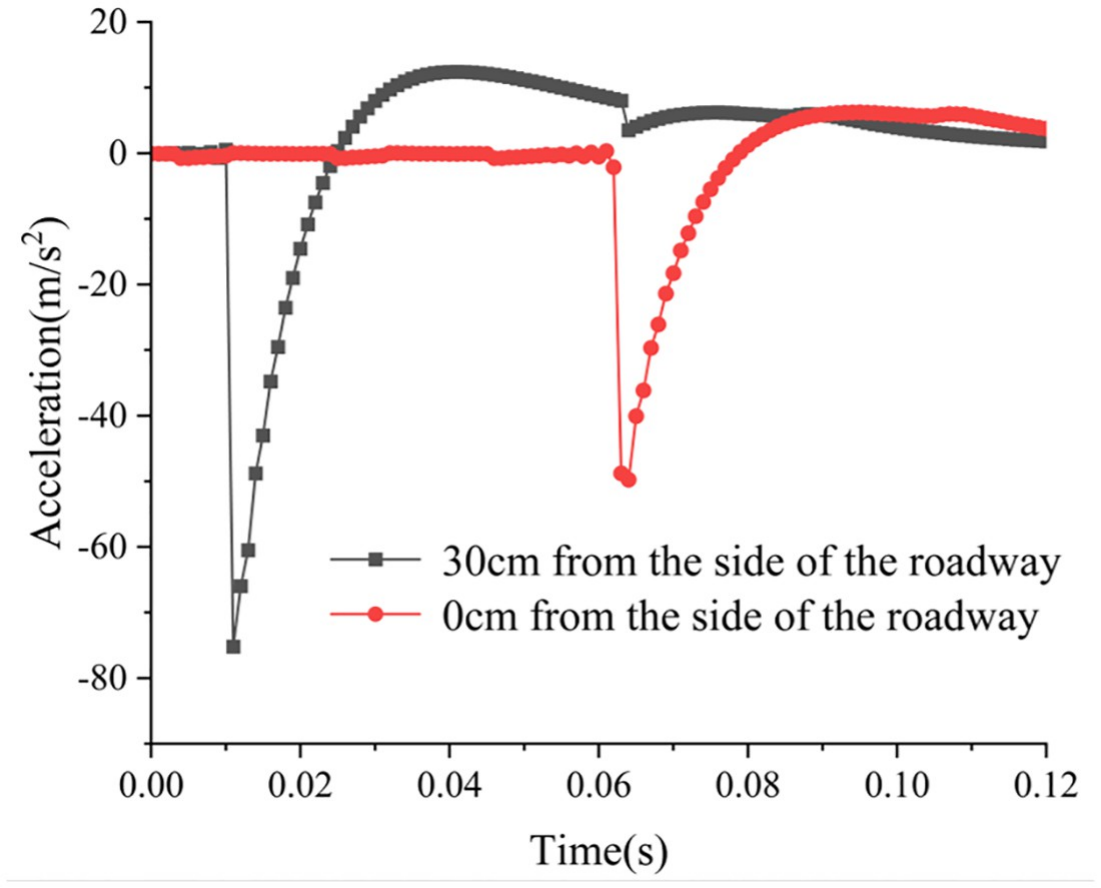
Acceleration curve diagram of variable diameter borehole test scheme.

Figs 22 and 23 depict the monitoring curves illustrating changes in acceleration waveforms for conventional boreholes and variable-diameter boreholes subjected to dynamic disturbance. Acceleration sensors are positioned both at the side of the roadway and 30 cm away from it (where positive acceleration denotes movement away from the roadway) to investigate how conventional and variable-diameter boreholes absorb and dissipate dynamic load acceleration transmission. Note that due to the dynamic strain gauge’s collection frequency of 100 Hz in the experiment, only 100 data points are extracted per second, limiting the available data. Consequently, the acceleration curve exhibits only a few peaks and troughs, deviating significantly from the ideal acceleration waveform curve. Despite being influenced by the sampling frequency, the acceleration peak remains consistent, thus not impacting the final experimental outcomes. In the scenario of conventional borehole, the acceleration sensor records a maximum acceleration of 75.21 m/s^2^ at the roadway’s side and 49.76 m/s^2^ 30 cm away, indicating a decrease of 33.84%. In contrast, with variable diameter borehole, the acceleration sensor records a maximum acceleration of 75.95 m/s^2^ at the roadway’s side and 32.21 m/s^2^ 30 cm away, indicating a decrease of 57.59%. The reduction in acceleration due to variable diameter borehole exceeds that of conventional borehole by 70.18%. The rationale is analyzed as follows: employing a dumbbell as the dynamic load disturbance source generates a spherical stress wave upon impact with the model, which then propagates radially from the disturbance center to the periphery. The diffusion effect of the stress wave attenuates its strength, acceleration, velocity, and energy during propagation. Borehole through the rock and soil mass above the borehole results in the release of stress from the upper surrounding rock and loosening of the soil, thereby enhancing the absorption and dissipation of the dynamic load stress wave. Overall, conventional boreholes are relatively small, limiting their capacity for absorbing and dissipating dynamic load stress waves. Conversely, the significant deep reaming of variable diameter boreholes results in a higher degree of stress release in the surrounding rock above the borehole, leading to loosening of the soil and greater absorption and dissipation of the dynamic load stress wave. Consequently, boreholes in the variable diameter borehole test scheme exhibit a more substantial reduction in dynamic load acceleration.

## 5. Conclusion

This study focuses on a typical rock burst coal mine as the engineering context, investigating how variable diameter borehole parameters (deep reaming diameter, depth, and spacing) affect stress, dissipation energy density, and the transmission of dynamic load stress waves in the surrounding rock of roadways through theoretical analysis, numerical simulation, and similar simulation testing. The study yields the following conclusions:

1. The stress at any point in the coal surrounding the variable diameter borehole correlates with the borehole radius, lateral pressure coefficient, and distance from the point to the borehole center. The range of plastic zone is related to the diameter, spacing and depth of borehole.
2. With larger diameters and smaller spacing of deep reaming, stress concentration in the surrounding rock of the roadway shifts deeper, potentially forming stress double peak areas, resulting in greater absorption and dissipation of dynamic load stress waves. The depth variation of deep reaming has minimal impact on the pressure relief and transmission of dynamic load stress waves in roadway surrounding rock due to overall borehole diameter limitations. Optimal pressure relief occurs when the variable diameter position is within the vertical stress peak position of the unrelieved roadway surrounding rock.
3. This study examines the disturbance patterns of variable-diameter borehole on the stability of roadway surrounding rock from an energy dissipation perspective. The findings suggest that when the depth of deep reaming is less than 16 meters, pressure relief borehole has minimal impact on the energy dissipation concentration density in the surrounding rock of the roadway. At this depth, the zone of variable-diameter borehole is situated deep within the surrounding rock of the roadway, where the rock’s low elastic energy reserves lead to limited dissipated energy. As the depth of deep reaming increases, exceeding 16 meters, the dissipated energy density in the pressure relief zone of roadway surrounding rock gradually stabilizes. It is determined that the variable diameter position should be located within the original stress peak position in the surrounding rock of the roadway.

## Supporting information

S1 Data (ZIP)
